# DeST-OT: Alignment of Spatiotemporal Transcriptomics Data

**DOI:** 10.1101/2024.03.05.583575

**Published:** 2024-03-10

**Authors:** Peter Halmos, Xinhao Liu, Julian Gold, Feng Chen, Li Ding, Benjamin J. Raphael

**Affiliations:** 1 Department of Computer Science, Princeton University, 35 Olden St, Princeton, NJ 08544; 2 Center for Statistics and Machine Learning, Princeton University, 26 Prospect Ave, Princeton, NJ 08544; 3 Departments of Medicine and Genetics, Siteman Cancer Center, Washington University in St. Louis, St. Louis, MO 63110; 4 McDonnell Genome Institute, Washington University in St. Louis, St. Louis, MO 63108

## Abstract

Spatially resolved transcriptomics (SRT) measures mRNA transcripts at thousands of locations within a tissue slice, revealing spatial variations in gene expression and distribution of cell types. In recent studies, SRT has been applied to tissue slices from multiple timepoints during the development of an organism. Alignment of this *spatiotemporal* transcriptomics data can provide insights into the gene expression programs governing the growth and differentiation of cells over space and time. We introduce DeST-OT (**De**velopmental **S**patio**T**emporal **O**ptimal **T**ransport), a method to align SRT slices from pairs of developmental timepoints using the framework of optimal transport (OT). DeST-OT uses *semi-relaxed* optimal transport to precisely model cellular growth, death, and differentiation processes that are not well-modeled by existing alignment methods. We demonstrate the advantage of DeST-OT on simulated slices. We further introduce two metrics to quantify the plausibility of a spatiotemporal alignment: a *growth distortion metric* which quantifies the discrepancy between the inferred and the true cell type growth rates, and a *migration metric* which quantifies the distance traveled between ancestor and descendant cells. DeST-OT outperforms existing methods on these metrics in the alignment of spatiotemporal transcriptomics data from the development of axolotl brain.

## 1 Introduction

Spatially Resolved Transcriptomics (SRT) technologies [37, 33, 28] measure gene expression simultaneously from thousands of cells or spots from a tissue slice, linking the gene expression measurement to the physical location within the tissue. These technologies enable exploration of tissue organization by analyzing cells within their native microenvironment, opening the door to the study of spatial biology [1, 17]. In some cases, SRT is applied to multiple slices from the same tissue. Joint analysis of multi-slice spatial data helps with the data sparsity problem in individual slices, enabling downstream analyses such as 3D differential expression or 3D cell-cell communication [23]. Multiple methods have been developed for alignment of multi-slice SRT data. For example, PASTE [45] integrates multiple slices from the same tissue and reconstructs the tissue gene expression in 3D, and PASTE2 [24] extends PASTE to partially overlapping slices. STalign [8] is an image registration method finding a diffeomorphism between the H&E images of two spatial slices. GPSA [18] uses Gaussian processes to register spatial slices onto a common coordinate system, while SLAT [44] relies on graph neural networks and adversarial learning.

Another recent exciting application is to apply SRT to tissues taken from multiple timepoints of a developmental process [5]. Alignment of slices from multiple timepoints can provide insights into the gene expression programs governing the growth and differentiation of cells over space and time. However, alignment of spatiotemporal transcriptomics data presents unique challenges as there is a complicated interplay between proliferation and apoptotic cell dynamics in the sculpting of developing tissue [42]. Regions of the tissue may grow or shrink, creating many-to-one relationships between the spots from consecutive timepoints. Cells also change in gene expression and differentiate into new cell types during development. Moreover, slices no longer come from the same batch (individual or time-point) and thus may exhibit batch effects.

The existing methods for temporal alignment of single-cell data or for spatiotemporal alignment suffer from important limitations. Waddington-OT [34] aligns temporal single cell data for reprogramming datasets, but does not take spatial information into account. A recent preprint [21] describes moscot, a method that relaxes the OT formulation in PASTE to use *unbalanced optimal transport* [36]. moscot allows for cell growth and death using a curated set of proliferation and apoptosis genes as prior knowledge but optimizes an objective function that encourages static shape-matching. Other works [18, 44] do not necessarily quantify growth, and often have methodological and robustness limitations.

We introduce DeST-OT, a method to align spatiotemporal transcriptomics data that consists of SRT slices from multiple timepoints. DeST-OT proposes a novel *semi-relaxed* optimal transport framework, leading to unsupervised discovery of cell growth and apoptosis without relying on existing gene annotations. DeST-OT aligns differentiating cells along a manifold jointly defined by transcriptomic information and spatial information, leading to both biologically and physically valid alignments. DeST-OT accounts for spatiotemporal scenarios by modeling three orders of interactions between cells in a developing tissue, bridging a gap in the standard Fused Gromov-Wasserstein (FGW) OT objective [38].

We demonstrate the advantages of DeST-OT on both simulated spatiotemporal data and spatiotemporal data from axolotl brain development. To evaluate the performance of different methods we introduce the *growth distortion metric* quantifying the accuracy of the inferred cell growth within a tissue across timepoints, and the *migration metric* quantifying the distance that cells migrate during development under an alignment. We show that DeST-OT produces alignments that are more growth-aware on simulated data. DeST-OT alignments are more biologically realistic in terms of the growth inferred and the distance cells migrate compared to other methods. DeST-OT infers biologically valid cell type transitions on a spatiotemporal dataset of axolotl brain providing insights into the growth dynamics of brain development.

## 2 Methods

### 2.1 Formulation

A spatially resolved transcriptomics slice is represented by a tuple $𝒮=\left(X,S\right)$. $X\in {\mathbb{N}}^{n\times p}$ is the transcript count matrix where each row $x_{r}$ is the gene expression vector of the corresponding spot, n the number of spots and p the number of genes measured. $S\in {\mathbb{R}}^{n\times 2}$ is the spatial position matrix, where the rows ${\text{s}}_{r}$ encode the $\left(x,y\right)$ coordinates of each spot. Given slices ${𝒮}_{1}=\left(X^{\left(1\right)},S^{\left(1\right)}\right)$ and ${𝒮}_{2}=\left(X^{\left(2\right)},S^{\left(2\right)}\right)$, measured on the same set of genes at timepoints $t_{1}$ and $t_{2}$, the goal is to derive an alignment matrix $\text{Π}\in {\mathbb{R}}_{+}^{n_{1}\times n_{2}}$, whose entry $\Pi_{ij}$ is positive and gives the probability that the cell(s) in spot i of ${𝒮}_{1}$ are the progenitors of the cell(s) in spot j of ${𝒮}_{2}$. The probabilities in the alignment matrix Π are derived to minimize some cost based on the gene expression at each spot and the spatial locations of aligned spots.

We use the mathematical tool of optimal transport (OT) to solve for Π. OT finds the most efficient way of moving mass between two distributions [30], and has previously been applied to single-cell alignment [11, 40] and spatial transcriptomics alignment [24, 45]. We seek to transport the mass of slice ${𝒮}_{1}$ to ${𝒮}_{2}$ where the mass is represented as a distribution over each slice’s spots. In the spatiotemporal setting, the amount of mass transferred between a spot at an earlier timepoint and a spot at a later one indicates how probable it is that the former spot is the ancestor of the latter. PASTE [45] uses OT to solve a related problem of static (non-temporal) spatial alignment, in which ${𝒮}_{1}$ and ${𝒮}_{2}$ are adjacent slices of the same tissue from the same timepoint, and minimizes the following objective function:

(1) $${ℰ}_{\text{PASTE }}\left(\Pi \right)=\left(1-\alpha \right)\sum_{i,j^{\prime }}\;C_{ij^{\prime }}\Pi_{ij^{\prime }}+\alpha \sum_{i,j^{\prime },k,l^{\prime }}\;{\left(D_{ik}^{\left(1\right)}-D_{j^{\prime }l^{\prime }}^{\left(2\right)}\right)}^{2}\Pi_{ij^{\prime }}\Pi_{kl^{\prime }}.$$

This objective function is a convex combination of two terms weighted by a balance parameter α. The first term, which we call the *feature term*, encourages matching spots with similar gene expression, and is also called the Wasserstein term in the OT literature [30]. We use the convention that a prime on an index, e.g. j′, refers to a spot in the second slice, while the absence of a prime denotes a spot in the first slice. The ij′-th entry of the matrix $C\in {\mathbb{R}}^{n_{1}\times n_{2}}$ is the distance in expression space between expression vector $x_{i}$ at ${𝒮}_{1}$-spot i and expression vector $x_{j^{\prime }}^{\prime }$ at ${𝒮}_{2}$-spot $j^{\prime }$. The second term, which we call the *spatial term*, encourages matching the intra-slice spatial distance between pairs of spots in each slice, and is called the Gromov-Wasserstein (GW) term [26, 31]. Matrices $D^{\left(1\right)}$ and $D^{\left(2\right)}$ are defined by intra-slice spatial distances $D_{ij}={\left\|s_{i}-s_{j}\right\|}_{2}$. The convex combination of the feature term and the spatial term is called the *Fused Gromov-Wasserstein* (FGW) objective [38]

**Figure 1:**
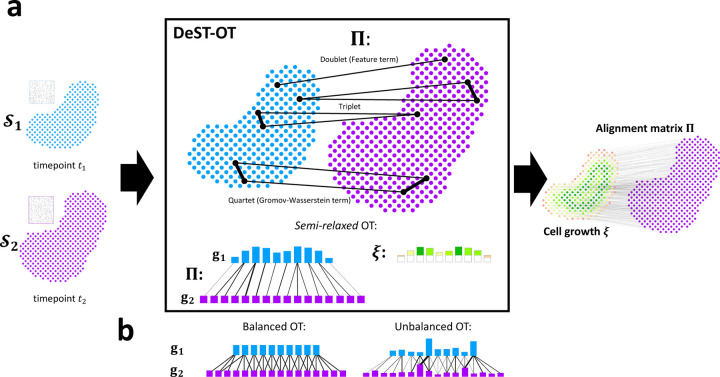
Overview of DeST-OT and semi-relaxed optimal transport (OT). (a) Given a pair of ${𝒮}_{1}$ and ${𝒮}_{2}$ from timepoints $t_{1}$ and $t_{2}$, respectively, DeST-OT infers an alignment matrix Π and a growth vector $\xi =\Pi 1_{n_{2}}-g_{1}$ by solving a semi-relaxed optimal transport problem with doublet, triplet, quartet objective costs. Green entries in ξ indicate cell growth while red entries indicate death (b) Balanced OT, which fixes both marginals $g_{1}$, $g_{2}$, and unbalanced OT, which varies both marginals.

PASTE optimizes (1) subject to the following constraints:

(2) $$\begin{array}{ll} \text{min} & {ℰ}_{\text{PASTE }}\left(\Pi \right)\\ \;s.t.\; & \Pi 1_{n_{2}}=g_{1},\;\Pi^{T}1_{n_{1}}=g_{2},\;\Pi \geq 0 \end{array}$$

where $g_{1}\in {\mathbb{R}}^{n_{1}}$, $g_{2}\in {\mathbb{R}}^{n_{2}}$ are uniform probability measures supported on the indices $i\in \left\{1,\ldots ,n_{1}\right\}$ and $j^{\prime }\in \left\{1,\ldots ,n_{2}\right\}$ **1** is a vector of all one’s. These constraints are called *balanced* optimal transport (OT) (Fig. 1b) because the alignment matrix satisfies the marginals $g_{1}$, $g_{2}$ strictly.

A recent preprint [21] introduced moscot, a modification of PASTE to use *unbalanced* OT, which is suggested to be helpful for spatiotemporal alignment. Specifically, moscot removes the equality constraints on the marginals, $\Pi {\text{1}}_{n_{2}}=g_{1}$, $\Pi^{T}{\text{1}}_{n_{1}}=g_{2}$ of (2), replacing these with two soft constraints in the form of Kullback-Leibler (KL) divergences:

(3) $$\begin{array}{ll} \text{min} & {ℰ}_{\text{PASTE}}\left(\Pi \right)+\frac{\epsilon \tau_{a}}{1-\tau_{a}}\text{KL}\left(\Pi 1_{n_{2}}∥g_{1}\right)+\frac{\epsilon \tau_{b}}{1-\tau_{b}}\text{KL}\left(\Pi^{T}1_{n_{1}}∥g_{2}\right)-\epsilon \text{H}\left(\Pi \right)\\ \;s.t.\; & \Pi \geq 0. \end{array}$$

Here $\tau_{a}$, $\tau_{b}\in \left(0,1\right)$ are hyperparameters determining the penalty for the marginals deviating from $g_{1}$ and $g_{2}$. $\text{H}\left(\cdot \right)$ is the entropy, where $\text{H}\left(\Pi \right)=-\sum_{i,j^{\prime }}\Pi_{ij^{\prime }}\left(\text{log}\left(\Pi_{ij^{\prime }}\right)-1\right)$. This entropic regularization accelerates the optimization of Π [9]. moscot has multiple limitations for spatiotemporal alignment. First, the method is supervised: to account for cell growth and death, moscot adjusts $g_{1}$ over the first slice using the expression of a predefined set of marker genes, limiting its applicability to organisms and tissues with good prior knowledge. Secondly, the fully unbalanced formulation allows mass to shift around both marginals, limiting the interpretability of the growth information (Supplement § S1.4). Third, the spatial term is not amenable to tissue expansion because it prefers to align identical shapes: ${\left(D_{ik}^{\left(1\right)}-D_{j^{\prime }l^{\prime }}^{\left(2\right)}\right)}^{2}\Pi_{ij^{\prime }}\Pi_{kl^{\prime }}$ is minimized when the distances inside the square are the same.

DeST-OT uses *semi-relaxed* optimal transport (Fig. 1a) and optimizes a growth-aware objective function modeling different levels of interactions between cells, capturing the growth dynamics comprehensively. In the *semi-relaxed* optimal transport framework (Fig. 1), we relax only the constraint $\Pi {\text{1}}_{n_{2}}=g_{1}$ in (2), replacing it by a KL divergence in the objective function, while the other constraint $\Pi^{T}{\text{1}}_{n_{1}}=g_{2}$ is kept. This ensures all of the spots in the second slice are mapped to from the first, while spots in the first slice can contribute a different amount of mass depending on whether they are growing or dying. We set both $g_{1}$, $g_{2}$ to assign equal weight $\frac{1}{n_{1}}$ to each spot. That is, $g_{1}$ is the uniform probability measure over spots in ${𝒮}_{1}$, while $g_{2}$ is a *positive* measure over spots in ${𝒮}_{2}$. We define an interpretable *growth vector* ξ that represents a mass-flux across the two timepoints indicating the magnitude of growth and death for each spot in ${𝒮}_{1}$,

(4) $$\xi =\Pi 1_{n_{2}}-g_{1}.$$

The growth vector $\xi \in {\mathbb{R}}^{n_{1}}$ is the change in mass relative to a uniform prior $g_{1}$ at each spot. The total sum of the entries of ξ is therefore $\frac{n_{2}-n_{1}}{n_{1}}$, fixed in proportion to the total change of mass across slices. For a spot i at time $t_{1}$, $\xi_{i}>0$ means that spot i has *>* 1 descendant in the second slice, and correspondingly, $\xi_{i}<0$ implies spot i has *<* 1 descendant in the second slice. The growth vector ξ is a change in mass over time – to convert this into a growth rate, one can take $J=\text{log}\left(1+n_{1}\xi \right)/\left(t_{2}-t_{1}\right)$ (Supplement § S2). See Supplement § S1.4 and § S3 for further discussion of the growth vector ξ.

DeST-OT optimizes a growth-aware objective function subject to the semi-relaxed constraints. The objective cost of DeST-OT for finding an optimal spatiotemporal alignment matrix Π consists of three terms: a doublet term, a triplet term, and a quartet term. The doublet term, $\sum_{i,j^{\prime }}C_{ij^{\prime }}\Pi_{ij^{\prime }}$, is the same as the feature term in PASTE, where $C\in {\mathbb{R}}^{n_{1}\times n_{2}}$ is an inter-slice gene expression distance matrix. The term compares the expression of two spots, one from each slice, hence we call it the *doublet* term of our objective.

The *quartet* term compares spot-pairs, with one pair from each slice. The quartet term is defined as $\sum_{i,j^{\prime },k,l^{\prime }}{\left(M_{ik}^{\left(1\right)}-M_{j^{\prime }l^{\prime }}^{\left(2\right)}\right)}^{2}\Pi_{ij^{\prime }}\Pi_{kl^{\prime }}$, where each matrix $M^{\left(i\right)}$ is defined to be the entrywise product of the square matrices $C^{\left(i\right)}$ and $D^{\left(i\right)}$. $C^{\left(i\right)}\in {\mathbb{R}}^{n_{i}\times n_{i}}$ is the distance in the expression space between each pair of spots on slice i. $D^{\left(i\right)}$ is the intra-slice spatial distance matrix as in PASTE. The quartet term is equivalent to the spatial (GW) term of both PASTE and moscot but with matrices $M^{\left(i\right)}$ that jointly model transcriptomic and spatial information, and with the semi-relaxed constraint applied to the gradient (Supplement § S4). We refer to M as *merged feature-spatial matrices*. While the spatial distance matrices ($D^{\left(1\right)}$, $D^{\left(2\right)}$) are appropriate for static alignment, they encode a rigid geometry that does not account for spatial deformations accompanying growth. On the contrary, DeST-OT matches a more flexible feature-smoothed geometry between the two slices, accounting for expansion or shrinkage of tissues during development.

The *triplet* term models the ancestor-descendant relationship between a single ancestor spot and multiple descendent spots, an essential relationship in growing tissues that is not well modeled by the other terms. When an ancestor spot differentiates into multiple descendant spots, these descendant spots should be close to each other in both physical space and feature space. Correspondingly, multiple ancestors should also be close. We make this notion precise by adding a *triplet* term to our objective: ${ℰ}_{\text{triplet }}\left(\Pi \right)=\sum_{ij^{\prime }k^{\prime }}\Pi_{ij^{\prime }}\Pi_{ik^{\prime }}M_{j^{\prime }k^{\prime }}^{\left(2\right)2}+\sum_{ijk^{\prime }}\Pi_{ik^{\prime }}\Pi_{jk^{\prime }}M_{ij}^{\left(1\right)2}$. The entrywise squares on the M matrices match the form of the quartet term, upweighting the triplet summands which enforce the similarity of descendants and ancestors. Adding these terms to our objective function has a regularizing effect: Π is penalized for predicting distant descendants $j^{\prime }$, $k^{\prime }$ of the same spot i in the first slice, or for predicting distant ancestors i, j of the same spot $k^{\prime }$ in the second slice. Distance is interpreted to be both spatial and transcriptomic due to the merged feature-spatial M matrices.

We call the sum ${ℰ}^{\text{M}}$ of the triplet term and the quartet term the *merged feature-spatial* term, since both use merged feature-spatial matrices and encourage alignments to respect developmental dynamics in both physical space and gene expression space. Specifically, we define

(5) $${ℰ}^{M}\left(\Pi \right)=\frac{1}{2}\left(\underset{\text{triplet }}{\underset{︸}{\sum_{i,j^{\prime },k^{\prime }}\;\Pi_{ij^{\prime }}\Pi_{ik^{\prime }}M_{j^{\prime }k^{\prime }}^{\left(2\right)2}+\sum_{i,j,k^{\prime }}\;\Pi_{ik^{\prime }}\Pi_{jk^{\prime }}M_{ij}^{\left(1\right)2}}}+\underset{\text{quartet}}{\underset{︸}{\sum_{i,j^{\prime },k,l^{\prime }}{\left(M_{ik}^{\left(1\right)}-M_{j^{\prime }l^{\prime }}^{\left(2\right)}\right)}^{2}\Pi_{ij^{\prime }}\Pi_{kl^{\prime }}}}\right).$$

Combining all of the above, the DeST-OT objective function is:

(6) $${ℰ}_{\text{DeST-OT }}=\left(1-\alpha \right)\underset{\text{doublet}}{\underset{︸}{\sum_{i,j^{\prime }}\;C_{ij^{\prime }}\Pi_{ij^{\prime }}}}+\alpha {ℰ}^{M}\left(\Pi \right)$$

The combination of the doublet, triplet, and quartet terms captures lower to higher order of interactions between spots in a growing tissue (Fig. 1a). The DeST-OT optimization problem, with entropic regularization and the semi-relaxed constraints is

(7) $$\begin{array}{ll} \text{ min} & {ℰ}_{\text{DeST}-\text{OT}}\left(\Pi \right)+\gamma \text{KL}\left(\Pi {\text{1}}_{n_{2}}∥g_{1}\right)-\epsilon \text{H}\left(\Pi \right)\\ \;s.t. & \Pi^{T}{\text{1}}_{n_{1}}=g_{2},\;\Pi \geq 0 \end{array}$$

The balance parameter α balances the contribution of the feature term and the merged feature-spatial term to the alignment. γ governs the compliance of the semi-relaxed constraint, and ϵ governs the strength of entropic regularization. We discuss the effect of these hyperparameters in the Results section.

### 2.2 Optimization Using Sinkhorn

We solve the DeST-OT optimization problem by deriving a variant of the Sinkhorn algorithm, which has become the canonical way to compute OT alignments due to its speed [9]. One may convert an optimal transport problem with a general objective into the framework of Sinkhorn by adding an entropy regularization $\text{-}\epsilon \text{H}\left(\text{Π}\right)$ to the objective function. Following the framework of Sinkhorn, the DeST-OT optimization problem (7) includes an entropy regularization term and we derive a set of updates from the KKT conditions for the semi-relaxed constraints to solve for Π. In practice, we take the dual of the semi-relaxed optimization to convert these updates into the log-domain [35], avoiding numerical overflow. The details of the optimization procedure are discussed in Supplement § S4.

### 2.3 Assessing alignment quality by cellular growth and migration

The true spatiotemporal alignment is often unknown, making it difficult to evaluate the accuracy of an alignment. We introduce two metrics to quantify the plausibility of an alignment: the *growth distortion metric* and the *migration metric*. The growth distortion quantifies the difference between the inferred growth and the proportional change of cell types in the two slices, given cell type labels for each spot in both slices. The migration metric quantifies how far cells “move” from the first timepoint to the second in a common coordinate framework describing the actual tissue. We say that an alignment Π is biologically valid when its growth distortion is ≈ 0, and physically valid if the migration distance is low.

#### 2.3.1 A metric of growth distortion

Given an alignment matrix Π, we define the *growth distortion metric* to quantify how well the growth vector ξ defined by equation (4) matches the observed change in proportion of given cell type labels over both slices. Formally, we are given a partition ${𝒫}_{1}={\left({𝒫}_{1}\left(p\right)\right)}_{p=1}^{P}$ of spots in slice 1, where the set ${𝒫}_{1}\left(p\right)$ consists of all spots of cell type p at time $t_{1}$, and a partition ${𝒫}_{2}={\left({𝒫}_{2}\left(p\right)\right)}_{p=1}^{P}$ of spots at timepoint $t_{2}$. The mass $m_{1}\left(p\right)$ of cell type p at time $t_{1}$ is $m_{1}\left(p\right)=\left|{𝒫}_{1}\left(p\right)\right|$, the number of $t_{1}$-spots with the label p. Likewise, the mass $m_{2}\left(p\right)$ of cell type p at time $t_{2}$ is $m_{2}\left(p\right)=\left|{𝒫}_{2}\left(p\right)\right|$. The change-in-mass for cell type p across these two timepoints is then $m_{2}\left(p\right)-m_{1}\left(p\right)$. To ensure that the mass change has the same scale as the growth vector ξ, we normalize the change-in-mass as $\frac{m_{2}\left(p\right)-m_{1}\left(p\right)}{n_{1}}$ since DeST-OT marginals assign mass $\frac{1}{n_{1}}$ to each spot, while the counting measure used to define the $m_{t}\left(p\right)$ assigns mass 1 to each spot.

We define the growth distortion metric under two assumptions: first, that there are no cell type transitions between distinct cell types (we discuss how to relax this assumption shortly). Second, the burden of accomplishing the change in mass is shared equally across cells of the same type. This second assumption can be viewed as an entropy-maximizing assumption. Under these two assumptions, the “true” growth $\gamma \left(p\right)$ at any $i\in {𝒫}_{1}\left(p\right)$ is

(8) $$\gamma \left(p\right)=\frac{1}{m_{1}\left(p\right)}\left(\frac{m_{2}\left(p\right)-m_{1}\left(p\right)}{n_{1}}\right).$$

Note that summing these values over all $t_{1}$-spots yields $\frac{n_{2}-n_{1}}{n_{1}}$, the total (normalized) change in mass across the two slices (Supplement § S5.1). The *growth distortion metric* ${𝒥}_{growth}$ of an alignment matrix Π with its associated ξ, relative to cell type partitions ${𝒫}_{1}$ and ${𝒫}_{2}$, measures the total distortion between the inferred growth ξ and the true growth γ at each spot:

(9) $${𝒥}_{growth\;}=\sum_{p=1}^{P}\;\sum_{i\in {𝒫}_{1}\left(p\right)}\;{\left\|\xi_{i}-\gamma \left(p\right)\right\|}_{2}^{2}.$$

We generalize the growth distortion metric to the case when cell type transitions are present (but unknown) using a reverse-time transition matrix $T\in {\mathbb{R}}^{P\times P}$. This matrix acts on a vector of cell type masses, redistributing the mass ${\text{m}}_{2}$ at time $t_{2}$ to the ancestral cell types at $t_{1}$ via the update $m_{1}=Tm_{2}$. To compute the growth distortion metric for an alignment matrix Π, we use the following cell type transition matrix T:

(10) $$T_{pq}=\left(\frac{n_{1}}{m_{2}\left(q\right)}\right)\sum_{i\in {𝒫}_{1}\left(p\right)}\;\sum_{j^{\prime }\in {𝒫}_{2}\left(q\right)}\;\Pi_{ij^{\prime }},$$

and prove in Proposition 2 of Supplement § S5.2 that the above T minimizes ${𝒥}_{growth}$ for a given Π across all T‘s. That is, when we do not know the true cell type transitions, we compute the growth distortion of an alignment as the lowest distortion it could possibly achieve under any cell type transition.

#### 2.3.2 A metric of cell migration

We introduce a migration metric ${𝒥}_{migration}$ of an alignment Π between two slices that quantifies the distance cells move under the alignment. This metric formalizes the intuition that the descendants of a cell tend to be close to their parent, particularly over short time intervals. Given an alignment Π and function $\varphi :{\mathbb{R}}^{2}\to {\mathbb{R}}^{2}$ that places slice ${𝒮}_{2}$ into a common-coordinate frame with slice ${𝒮}_{1}$, we define the *migration metric* as:

(11) $${𝒥}_{migration}=E_{\left(i,j^{\prime }\right)∼\Pi }\left[{\left\|s_{i}-\varphi \left(s_{j^{\prime }}\right)\right\|}_{2}^{2}\right],$$

namely the average squared distance between spatial coordinate $s_{i}$ in slice ${𝒮}_{1}$ and transformed second-slice spatial coordinate $\varphi \left(s_{j^{\prime }}\right)$ over pairs $\left(i,j^{\prime }\right)$ that are sampled proportionally to Π (column normalizing Π, as in Supplement Eq. (49)). In the results reported below, we use the function $\varphi \left(z\right)=Q\left(z-h\right)$ for an orthogonal transformation Q and translation vector $h\in {\mathbb{R}}^{2}$ that solve a generalized Procrustes’ problem (Supplement § S6). This describes a rigid-body transformation relating the coordinate frames of slice ${𝒮}_{1}$ and ${𝒮}_{2}$.

## 3 Results

### 3.1 Evaluation on simulated ST data

We evaluated DeST-OT and moscot on simulated data from one- and two-dimensional tissue slices with eight-dimensional feature expressions for each spot. For each timepoint, the feature at each spot varies within each cell type. Details of the simulation of features are in Supplement § S7. Since moscot assumes the marginals used as input to its OT problem are already adapted to cell proliferation and apoptosis using prior knowledge, we set moscot’s marginal over $t_{1}$ to account for changing cell type proportion in each experiment. DeST-OT uses semi-relaxed OT, and thus no prior knowledge of growth and death was required.

**Figure 2:**
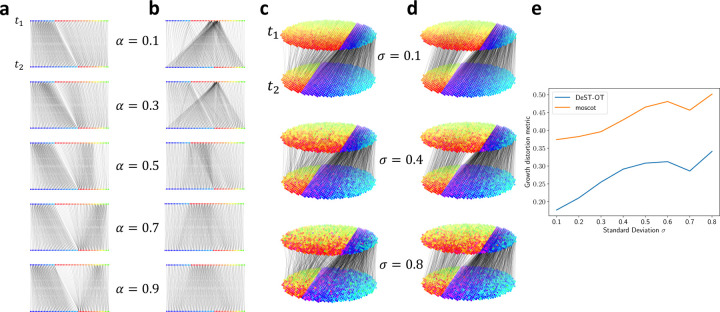
DeST-OT and moscot alignments on simulated data. **a**, DeST-OT and **b**, moscot alignment results for the balance parameter *α* ranging from 0.1 (mostly feature term) to 0.9 (mostly spatial terms), on 1D simulated slices. DeST-OT’s alignments indicate the cell type boundary, and color represents the polar angle made from two of the four non-zero coordinates in each cell type. **c**, DeST-OT and **d**, moscot alignments on 2D simulated slices with standard deviation of the expression noise *σ* = 0.1, 0.4, 0.8. Expression features are visualized similarly in 2D. **e**, The growth distortion metric for DeST-OT and moscot as a function of *σ*.

The first simulated dataset consisted of a pair of one-dimensional tissue slices, denoted as timepoint $t_{1}$ and $t_{2}$, each with 101 spots. There were two cell types across both slices. Slice $t_{1}$ had 30 spots of cell type A and 71 spots of cell type B; slice $t_{2}$ had 60 spots of cell type A and 41 spots of cell type B (Fig. 2ab). Therefore, cell type A grew by a factor of 2 from $t_{1}$ to $t_{2}$ and cell type B shrunk by roughly the same factor. We ran both DeST-OT and moscot on this pair of one-dimensional slices, varying the balance parameter *α* in the objective functions from *α* = 0.1 to *α* = 0.9, gradually placing more weight on each method’s spatial term (i.e. the merged feature-spatial term in DeST-OT, and the GW term in moscot) in the objective. We found that DeST-OT alignments are robust to varying *α*, always aligning cells to the correct cell types across timepoints (Fig. 2a). DeST-OT captures the true growth pattern of cells for all values of *α* because the merged feature-spatial term of DeST-OT incorporates both transcriptional and spatial information. On the other hand, moscot has greater difficulty capturing growing and shrinking cell types with larger *α*, as its spatial term emphasizes matching the shapes of the two slices as discussed in §2.1 (Fig. 2b). This demonstrates the effectiveness of DeST-OT’s spatiotemporal objective function, as well as the importance of DeST-OT’s semi-relaxed framework even when aligning slices with the same number of spots.

We next tested DeST-OT and moscot on a more realistic simulation with two-dimensional slices and feature expression noise. We generated two elliptical slices at $t_{1}$ and $t_{2}$ of the same size (988 spots), again with two cell types across the slices; cell type A occupies the right regions of the slices in (Fig. 2b), while cell type B is on the left. Slice $t_{1}$ had 240 spots of cell type A and 748 spots of cell type B; slice *t*_2_ had 726 spots of cell type A and 262 spots of cell type B. Each cell type is characterized by a pair ${\text{f}}_{x,\mathrm{right}}$, ${\text{f}}_{x,\mathrm{left}}$ of eight-dimensional feature vectors for the x-direction, and another pair ${\text{f}}_{y,\mathrm{top}}$, ${\text{f}}_{y,\mathrm{bottom}}$ of feature vectors for the *y*-direction. The feature at a given spot in each cell type is a convex combination $\text{f}\left(x,y\right)=\lambda_{x}{\text{f}}_{x,\text{ right }}+\left(1-\lambda_{x}\right){\text{f}}_{x,\text{ left }}+\lambda_{y}{\text{f}}_{y,\text{ top }}+\left(1-\lambda_{y}\right){\text{f}}_{y,\text{ bottom }}$_,bottom_ of the x-direction feature vectors the y-direction feature vectors. The coefficients $\lambda_{x}$, $\lambda_{y}$ are determined by the horizontal and vertical distance to the spot’s cell type boundary. This creates a consistent gradient of features within each cell type (Supplement § S7). For $\left(x_{1},y_{1}\right)\in S^{\left(1\right)}$, $\left(x_{2},y_{2}\right)\in S^{\left(2\right)}$ we have that if $\lambda_{x_{1}}^{A}=\lambda_{x_{2}}^{A}$ and $\lambda_{y_{1}}^{A}=\lambda_{y_{2}}^{A}$ then ${\text{f}}_{A}\left(x_{1},y_{1}\right)={\text{f}}_{A}\left(x_{2},y_{2}\right)$ and the two spots should be aligned between timepoints 1 and 2 (likewise for cell type B). We then added zero-centered Gaussian noise with standard deviation σ independently to each feature dimension, with σ ranging from 0.1 to 0.8 in increments of 0.1. In addition to supplying moscot with the ground-truth adapted $t_{1}$-marginal, we also set it to be fully unbalanced with $\tau_{a}$ = 0.99, $\tau_{b}$ = 0.999 as suggested by the tutorial for noisy data.

DeST-OT aligns ancestor cells to descendant cells correctly along the cell type feature gradients across timepoints (Fig. 2c), capturing cell growth and death. DeST-OT alignments are robust to noise as well, aligning cell types correctly even with added noise in spot features. While moscot produces straighter alignments (Fig. 2d) we found that it identifies the ancestors of cell type B at $t_{2}$ to come from a small, similarly shaped region of cell type A at $t_{1}$ even with perfect growth knowledge encoded in the marginal ${\text{g}}_{1}$. Rather than aligning along a gradient of spatially expanding features, moscot’s spatial term prefers to align identical shapes, and is not suited to aligning tissue slices which grow and deform over time. DeST-OT consistently infers more accurate cell development as quantitatively shown by the growth distortion metric (Fig. 2e).

### 3.2 Axolotl brain development

**Figure 3:**
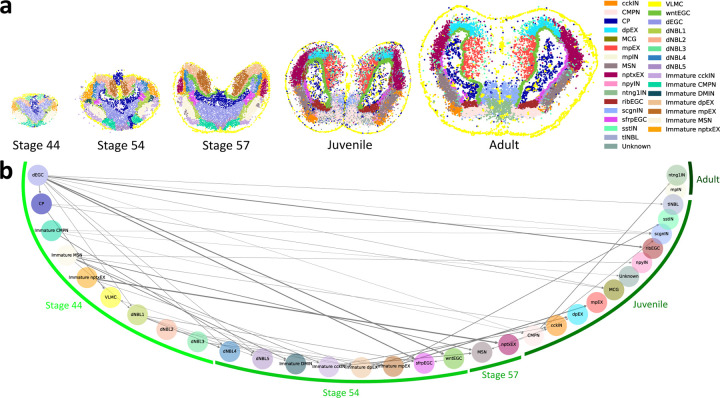
DeST-OT analysis of axolotl brain development. **a**, Stereo-seq data from axolotl brain sections at embryonic stage 44, embryonic stage 54, embryonic stage 57, Juvenile stage, and Adult stage, with cell type labels from [43] **b**, Cell type transition graph derived from DeST-OT alignments throughout axolotl brain development. The cell types are arranged in a half circle. A cell type is assigned to a developmental stage if it first appears in that stage. The width of the edges are proportional to the weight of transition. Self-loops are omitted.

**Figure 4:**
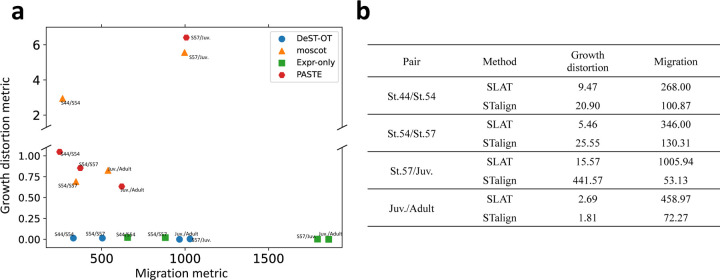
Alignment performance of various methods on the axolotl brain development dataset. **a**, The growth distortion metric and migration metric of DeST-OT, moscot, Expr-only, PASTE alignments on all pairs of axolotl brain development timepoints. **b**, The growth distortion and migration metric of SLAT and STalign alignments on all pairs of axolotl brain development timepoints. Not included in the plot in **a** because of high growth distortion.

We applied DeST-OT to analyze the developmental dynamics of the telencephalon, a region of the brain, in axolotl (*Ambystoma mexicanum*), a species of salamander. *Wei et. al.* [43] used Stereo-seq [5] to measure gene expression in the axolotl telencephalon at five development timepoints: three embryonic stages (44, 54, 57), Juvenile stage, and Adult stage (Fig. 3a). The slices grow in size, and progenitor cell types transition into mature cell types during the developmental process. We used DeST-OT, moscot, PASTE, STalign, and SLAT to infer an alignment between each pair of timepoints respectively, and computed the growth distortion and migration metric for each method (Fig. 4). Since new cell types appear at individual timepoints and the transitions between cell type during development are not annotated, we computed the growth distortion metric for each method under the cell type transition that minimizes their growth distortion as described in § 2.3. DeST-OT has the lowest growth distortion among all methods while maintaining low migration distances, demonstrating the quality of the DeST-OT alignments (Fig. 4). moscot and STalign achieve a low migration metric by shape-matching but have high growth distortions. SLAT has a low migration distance on this dataset as well, but cannot estimate cell growth and death accurately either.

We examined the cell type transition matrix T between each pair of adjacent timepoints derived from the DeST-OT alignment (§ 2.3) and the cell type annotations from [43] (Fig. S1). From these transition matrices, we derive a weighted directed graph showing all frequent transitions (*>*20%) (Fig. 3b). Many of the DeST-OT inferred cell type transitions are consistent with previously reported developmental trajectories. For example, we found that among all cell types, dEGCs (developmental ependymoglial cells) give rise to the largest number (11) of descendent cell types, consistent with previous studies which suggest that EGCs are equivalent to neural stem cells in mammals and contribute to neurogenesis during brain development [19, 20, 3]. Furthermore, immature cell types expressing early developmental markers disappear from the juvenile stage onward (Fig. 3a), and DeST-OT confirmed that immature cells of each cell type, such as CMPN or nptxEX, transition into their respective mature cell types. Previous studies suggested a potential lineage transition from EGCs to neuroblasts (NBLs) [29], and a transition from dEGC to NBL cell types that appear after stage 44 (dNBL4, dNBL5, tlNBL) is also found by DeST-OT. Finally, dEGCs disappear at stage 57, and DeST-OT predicts that it transitions mostly into ribEGCs located in the ventricular zone, which is the same spatial region where dEGCs located before and consistent with the findings in [43]. We observe a directed cycle between cckIN and MSN, probably because the two cell types are mixed together in the striatum region. There is no directed edge going into mpIN because it has multiple progenitor cell types and no cell type passes the threshold (20% in this case) for including an edge giving rise to mpIN (Fig. S1), indicating a diverse origin of mpIN.

**Figure 5:**
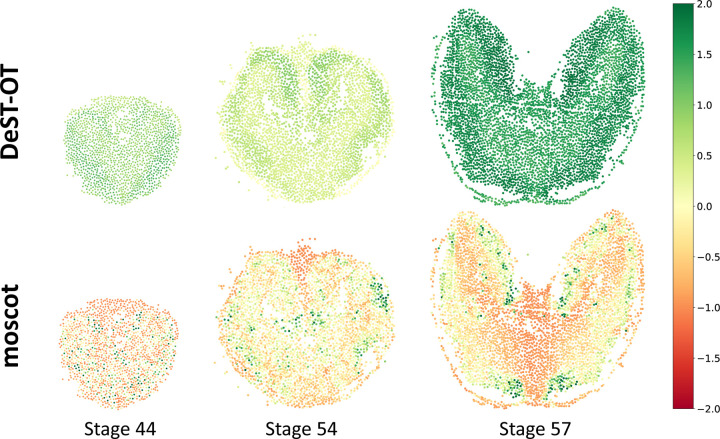
Growth patterns of axolotl brain. The growth of cells inferred by DeST-OT and moscot on the three embryonic stages of axolotl brain development. Growth vector ***ξ*** is normalized to unit of number of cells.

We found that the growth patterns of individual cells inferred by DeST-OT are more biologically reasonable than those inferred by moscot (Fig. 5). For the three embryonic stages, DeST-OT infers that all cells are growing and identifies differential growth patterns of tissue regions: the outer part of the telencephalon, mostly occupied by immature cell types, grows faster than the inner part. In contrast, the growth patterns inferred by moscot tend to be sparser, with a few “representative” cells have a high growth and thus a large number of descendants in the next timepoint, while most other cells are dying, which is not realistic for embryonic tissues. This is a computational artifact of the fully unbalanced OT formulation which allows a few “best” cells from the two slices to be aligned, hence does not fit spatiotemporal data where all descendant spots should be aligned.

## 4 Discussion

We introduce DeST-OT, a method for aligning spatiotemporal transcriptomics data and for inferring cell proliferation and apoptosis. Using a semi-relaxed optimal transport framework and an objective cost designed for spatiotemporal data, DeST-OT finds an alignment between progenitor and descendent cells in developmental spatial transcriptomics data, infers growth and death rates, and infers cell type transitions during tissue development. To quanitfy the performance of DeST-OT and other spatiotemporal alignment methods, we introduce the migration metric to quantify the distance cells travel under a spatiotemporal alignment and the growth distortion metric to quantify how accurately an alignment infers growth relative to ground-truth cell type annotations. We show on simulated data that DeST-OT outperforms other methods and infers cell growth and death accurately. We use DeST-OT to study axolotl brain development and confirm previously reported lineage transitions. DeST-OT alignments can elucidate developmental dynamics and may lay the ground for the discovery of their molecular basis. We also demonstrate that DeST-OT alignments are more biologically and physically realistic than competing methods.

Future work includes the evaluation of DeST-OT on other spatiotemporal datasets. There are currently few such publicly available datasets, but analysis of another unpublished dataset is ongoing and will be included in a future revision. DeST-OT will be a useful tool for biologists to gain new insights in spatiotemporal processes such as development and reprogramming, discovering new temporally and spatially dependent biological phenomena.

## Supplementary Material

Supplement 1
