# Supplementary material for: DeST-OT: Alignment of Spatiotemporal Transcriptomics Data
Source: bioRxiv. 2024 Mar 10:2024.03.05.583575. Preprint. [Version 1] doi: 10.1101/2024.03.05.583575 (PMC10942402; doi:10.1101/2024.03.05.583575)
Supplement: Supplement 1 [file NIHPP2024.03.05.583575v1-supplement-1.pdf]

bioRxiv preprint doi: https://doi.org/10.1101/2024.03.05.583575; this version posted March 10, 2024. The copyright holder for this preprint (which was not certified by peer review) is the author/funder, who has granted bioRxiv a license to display the preprint in perpetuity. It is made available under aCC-BY 4.0 International license.

Supplement

S1 Formulation

In vanilla optimal transport, and in the original formulation of PASTE [45], one has two constraints on alignment matrix $\mathbf{\Pi}$, given by probability measures $\mathbf{g}_1 \in \mathbb{R}^{n_1}, \mathbf{g}_2 \in \mathbb{R}^{n_2}$: the row-sum of $\mathbf{\Pi}$ must be $\mathbf{g}_1$, and the column-sum of $\mathbf{\Pi}$ must be $\mathbf{g}_2$. Measures $\mathbf{g}_1$ and $\mathbf{g}_2$ are supported on indices $i \in \{1, \ldots, n_1\}$ and $j' \in \{1, \ldots, n_2\}$, enumerating the spots of two slices of spatial transcriptomics data, $\mathcal{S}_1 = (\mathbf{X}^{(1)}, \mathbf{S}^{(1)})$ and $\mathcal{S}_1 = (\mathbf{X}^{(2)}, \mathbf{S}^{(2)})$. The rows $\mathbf{x}_i \equiv \mathbf{X}^{(1)}_{i,\cdot}$ of $\mathbf{X}^{(1)}$ and $\mathbf{x}'_{j'} \equiv \mathbf{X}^{(2)}_{j',\cdot}$ of $\mathbf{X}^{(2)}$ are indexed by spots, and each row is the expression vector at the given spot. Likewise, the rows $\mathbf{s}_i \equiv \mathbf{S}^{(1)}_{i,\cdot}$ of $\mathbf{S}^{(1)}$ and $\mathbf{s}'_{j'} \equiv \mathbf{S}^{(2)}_{j',\cdot}$ of $\mathbf{S}^{(2)}$ are the spatial coordinates of each spot. We use the convention that a prime on an index, e.g. j', refers to a spot in the second slice, while the absence of a prime on an index denotes a spot in the first slice.

S1.1 Context

In PASTE, a convex combination of standard inter-slice expression cost and a Gromov-Wasserstein cost are used (this combination referred to as *Fused Gromov-Wasserstein (FGW)*). The corresponding optimization problem is:

$$\begin{aligned} \min_{\mathbf{\Pi} \in \mathbb{R}_+^{n_1 \times n_2}} \quad & \left\{ (1-\alpha) \sum_{i,\, j'} \mathbf{C}_{ij'} \mathbf{\Pi}_{ij'} + \alpha \sum_{i,\, j',\, k,\, l'} \left(\mathbf{D}^{(1)}_{ik} - \mathbf{D}^{(2)}_{j'l'} \right)^2 \mathbf{\Pi}_{ij'} \mathbf{\Pi}_{kl'} \right\} \\ s.t. \quad & \mathbf{\Pi} \mathbf{1}_{n_2} = \mathbf{g}_1 = \frac{1}{n_1} \mathbf{1}_{n_1}, \quad \mathbf{\Pi}^T \mathbf{1}_{n_1} = \mathbf{g}_2 = \frac{1}{n_2} \mathbf{1}_{n_2}, \quad \mathbf{\Pi} \succcurlyeq 0 \end{aligned} \tag{12}$$

Above, $\mathbf{C} \in \mathbb{R}^{n_1 \times n_2}$ is the inter-slice (gene expression) feature-distance matrix $\mathbf{C}_{ij'} \equiv \mathbf{C}(\mathbf{x}_i, \mathbf{x}'_{j'})$ whose ij'-th entry is the distance in expression space between the expression vector $\mathbf{x}_i$ at spot i in the first slice and the expression vector $\mathbf{x}'_{j'}$ at spot j' in the second slice. Matrices $\mathbf{D}^{(1)}$ and $\mathbf{D}^{(2)}$ in (12) are intra-slice (spatial) distance matrices. Define the 4-tensor $\mathbf{L} \equiv \mathbf{L}(\mathbf{D}^{(1)}, \mathbf{D}^{(2)}) \in \mathbb{R}^{n_1 \times n_2 \times n_1 \times n_2}$ entry-wise via

$$\mathbf{L}_{i\, j'k\, l'} := \left(\mathbf{D}^{(1)}_{ik} - \mathbf{D}^{(2)}_{j'l'} \right)^2 \tag{13}$$

We let $\otimes$ denote the tensor-matrix multiplication operator, such that for the alignment matrix $\mathbf{\Pi} \in \mathbb{R}^{n_1 \times n_2}_{\geq 0}$, $\mathbf{L} \otimes \mathbf{\Pi}$ denotes the $n_1 \times n_2$ matrix whose ij'-th entry is $\sum_{k,\, l'} \mathbf{L}_{i\, j'k\, l'} \mathbf{\Pi}_{kl'}$. Let $\langle \cdot, \cdot \rangle_F$ denote the Frobenius inner product of matrices. In this notation, the objective function (12) can be reformulated as follows:

$$\begin{aligned} \min_{\Pi \in \mathbb{R}_+^{n_1 \times n_2}} \quad & \left\{ (1-\alpha) \Big\langle \mathbf{C}, \mathbf{\Pi} \Big\rangle_F + \alpha \Big\langle \mathbf{L} \otimes \mathbf{\Pi}, \mathbf{\Pi} \Big\rangle_F \right\} \\ s.t. \quad & \mathbf{\Pi} \mathbf{1}_{n_2} = \mathbf{g}_1 = \frac{1}{n_1} \mathbf{1}_{n_1}, \quad \mathbf{\Pi}^T \mathbf{1}_{n_1} = \mathbf{g}_2 = \frac{1}{n_2} \mathbf{1}_{n_2}, \quad \mathbf{\Pi} \succcurlyeq 0 \end{aligned} \tag{14}$$

with $\mathbf{L}$ as in (13). The only difference between PASTE2 [24] and the original PASTE is the inclusion of the constraint $\mathbf{1}_{n_1}^T \mathbf{\Pi} \mathbf{1}_{n_2} = s$, which goes along with a relaxation of the two marginal constraints to inequalities:

$$\begin{aligned} \min \quad & (1-\alpha) \Big\langle \mathbf{C}, \mathbf{\Pi} \Big\rangle_F + \alpha \Big\langle \mathbf{L} \otimes \mathbf{\Pi}, \mathbf{\Pi} \Big\rangle_F \\ s.t. \quad & \mathbf{\Pi} \mathbf{1}_{n_2} \leq \mathbf{g}_1 = \frac{1}{n_1} \mathbf{1}_{n_1}, \quad \mathbf{\Pi}^T \mathbf{1}_{n_1} \leq \mathbf{g}_2 = \frac{1}{n_2} \mathbf{1}_{n_2}, \quad \mathbf{1}_{n_1}^T \mathbf{\Pi} \mathbf{1}_{n_2} = s, \quad \mathbf{\Pi} \succcurlyeq 0 \end{aligned}$$

Note that by considering the dual problem, *partial optimal transport*, in which there is a constraint $s \in (0, 1)$ on the total mass $\mathbf{1}_{n_1}^T \mathbf{\Pi} \mathbf{1}_{n_2}$ transported, can be seen as an instance of unbalanced optimal transport associated to the total variation φ-divergence (see [36] § 4.2). *Unbalanced optimal transport* refers to the version of (14) obtained by deleting the two hard constraints, $\mathbf{\Pi} \mathbf{1}_{n_2} = \mathbf{g}_1$ and $\mathbf{\Pi}^T \mathbf{1}_{n_1} = \mathbf{g}_2$, on the marginals of $\mathbf{\Pi}$, and instead adding to the cost function in brackets a pair of "soft constraints" in the form of two terms: $\mathrm{D}_\varphi(\mathbf{\Pi} \mathbf{1}_{n_2} | \mathbf{g}_1) + \mathrm{D}_\varphi(\mathbf{\Pi}^T \mathbf{1}_{n_1} | \mathbf{g}_2)$. Here, $\varphi : (0, \infty) \to [0, +\infty]$ is a so-called *entropy function*, namely it is convex, positive, lower-semi-continuous, and with $\varphi(1) = 0$. Let $\mathrm{D}_\varphi(\cdot|\cdot)$ denote the associated φ-divergence, defined for positive measures α, β on some common space $\mathcal{X}$ as $D_\varphi(\alpha|\beta) = \int_{\mathcal{X}} \varphi(\frac{\mathrm{d}\alpha}{\mathrm{d}\beta}) \mathrm{d}\beta$, where we have supposed α and β are mutually absolutely continuous, for instance. When $\varphi(p) = p \log p - p + 1$, one recovers the *Kullback-Leibler (KL) divergence*, which we denote by $\mathrm{D}_\varphi(\cdot|\cdot) = \mathrm{KL}(\cdot\|\cdot)$ in this case.

bioRxiv preprint doi: https://doi.org/10.1101/2024.03.05.583575; this version posted March 10, 2024. The copyright holder for this preprint (which was not certified by peer review) is the author/funder, who has granted bioRxiv a license to display the preprint in perpetuity. It is made available under aCC-BY 4.0 International license.

S1.2 `DeST-OT` objective

Our view of an OT objective function as a *Hamiltonian*, assigning an energy to a given transport plan $\mathbf{\Pi}$, motivates the terminology we use for its components below. The first change we address in the formulation of `DeST-OT`, relative to `PASTE`, is the constraints on the optimization together with our calibration of mass on the second slice. Specifically, we relax the constraint $\mathbf{\Pi}\mathbf{1}_{n_2} = \mathbf{g}_1$, while preserving the other. This fixes the total mass transported by $\mathbf{\Pi}$ to be the total mass of the second (usually larger, in the spatiotemporal setting) slice, which is equipped with positive, *not necessarily probability*, measure $\mathbf{g}_2 = \frac{1}{n_1}\mathbf{1}_{n_2}$. This allows the $\mathbf{\Pi}$-marginal over the first slice, namely $\mathbf{\Pi}\mathbf{1}_{n_2}$, to be non-uniform, while fixing its total mass to the value $\frac{n_2}{n_1}$. One is naturally lead to introduce a *mass-flux term*,

$$\boldsymbol{\xi} = \mathbf{\Pi}\mathbf{1}_{n_2} - \mathbf{g}_1, \tag{15}$$

as this object becomes non-zero as soon as we relax the first marginal constraint. In section S1.4, we discuss the value of $\boldsymbol{\xi}$. The non-uniformity in $\mathbf{\Pi}\mathbf{1}_{n_2}$ reflects that not all spots in the first slice are necessarily contributing the same amount to spots in the second slice – different cell types may have different growth rates.

The second change we address concerns the matrices used by the Gromov-Wasserstein (GW) term $\mathcal{E}_{\mathrm{GW}}(\mathbf{\Pi}) = \langle \mathbf{L} \otimes \mathbf{\Pi}, \mathbf{\Pi} \rangle$. This term favors $\mathbf{\Pi}$ matrices which are nearly isometries between the two slices, as whenever $\mathbf{D}^{(1)}_{ik} = \mathbf{D}^{(2)}_{j'l'}$, the corresponding summand of the GW term $\mathcal{E}_{\mathrm{GW}}(\mathbf{\Pi})$ vanishes. For a more general pair of intra-slice cost matrices, (symmetric, non-negative matrices) $\mathbf{M}^{(1)} \in \mathbb{R}^{n_1 \times n_1}$ and $\mathbf{M}^{(2)} \in \mathbb{R}^{n_2 \times n_2}$, we generalize (13), defining the 4-tensor $\mathbf{L}^{\mathbf{M}} \equiv \mathbf{L}^{\mathbf{M}}(\mathbf{M}^{(1)}, \mathbf{M}^{(2)}) \in \mathbb{R}^{n_1 \times n_2 \times n_1 \times n_2}$ entry-wise via

$$\mathbf{L}^{\mathbf{M}}_{i\,j'\,k\,l'} := \left(\mathbf{M}^{(1)}_{ik} - \mathbf{M}^{(2)}_{j'l'}\right)^2, \tag{16}$$

and we define the analogous GW energy (using $\mathbf{M}^{(i)}$ in place of the $\mathbf{D}^{(i)}$). We also refer to it as a *quartet* energy, as each summand defining it corresponds to two pairs points:

$$\mathcal{E}^{\mathbf{M}}_{\mathrm{GW}}(\mathbf{\Pi}) \equiv \mathcal{E}^{\mathbf{M}}_{\mathrm{quartet}}(\mathbf{\Pi}) = \langle \mathbf{L}^{\mathbf{M}} \otimes \mathbf{\Pi}, \mathbf{\Pi} \rangle. \tag{17}$$

We define the matrices $\mathbf{M}^{(i)}$ from a pair of expression distance matrices for the first and second slice, $\mathbf{C}^{(1)} \in \mathbb{R}^{n_1 \times n_1}$, $\mathbf{C}^{(2)} \in \mathbb{R}^{n_2 \times n_2}$. These intra-slice feature matrices $\mathbf{C}^{(i)}$ are distinct from their inter-slice counterpart $\mathbf{C} \in \mathbb{R}^{n_1 \times n_2}_{+}$ which contains the distance in (expression) feature space from spots in slice 1 and slice 2, and is therefore not a symmetric, square matrix in general. We specifically choose $\mathbf{M}^{(i)}$ to be the Hadamard product of matrices $\mathbf{C}^{(i)}$ and $\mathbf{D}^{(i)}$, so that entry-wise, one defines $\mathbf{M}^{(1)}_{ik} = \mathbf{C}^{(1)}_{ik}\mathbf{D}^{(1)}_{ik}$ and $\mathbf{M}^{(2)}_{j'l'} = \mathbf{C}^{(2)}_{j'l'}\mathbf{D}^{(2)}_{j'l'}$. We refer to the $\mathbf{M}^{(i)}$ as *merged feature-spatial matrices*.

To $\mathcal{E}^{\mathbf{M}}_{\mathrm{GW}}$, we add a symmetric pair of terms $\mathcal{E}^{\mathbf{M}}_{\mathrm{triplet}^{(1)}}$ and $\mathcal{E}^{\mathbf{M}}_{\mathrm{triplet}^{(2)}}$ of the form

$$\begin{aligned} \mathcal{E}^{\mathbf{M}}_{\mathrm{triplet}^{(1)}}(\mathbf{\Pi}) &= \mathrm{Tr}\left[\mathbf{\Pi}\mathbf{V}^{(2)}\mathbf{\Pi}^T\right] = \sum_{i,j',k'} \mathbf{\Pi}_{ij'}\mathbf{\Pi}_{ik'}\mathbf{V}^{(2)}_{j'k'}, \\ \mathcal{E}^{\mathbf{M}}_{\mathrm{triplet}^{(2)}}(\mathbf{\Pi}) &= \mathrm{Tr}\left[\mathbf{\Pi}^T\mathbf{V}^{(1)}\mathbf{\Pi}\right] = \sum_{i,\,j,\,k'} \mathbf{\Pi}_{ik'}\mathbf{\Pi}_{jk'}\mathbf{V}^{(1)}_{ij} \end{aligned} \tag{18}$$

When $\mathbf{V}^{(2)} = (\mathbf{M}^{(2)})^2$, i.e. entry-wise squaring of the merged feature-spatial matrix, which is why the superscript indicates the same dependence on the $\mathbf{M}$-matrices as the GW term. Note that $\mathcal{E}^{\mathbf{M}}_{\mathrm{triplet}^{(1)}}$ is identical to the terms in the sum defining $\mathcal{E}^{\mathbf{M}}_{\mathrm{GW}}$ which have $i = k$, which is why we name it for the first slice instead using the index of the merged feature-spatial matrix involved in the definition. This energy $\mathcal{E}^{\mathbf{M}}_{\mathrm{triplet}^{(1)}}$ favors $\mathbf{\Pi}$ which predict a localized (in space, and in gene expression space) set of possible ancestors for each spot in the second slice.

Likewise, when $\mathbf{V}^{(1)} = (\mathbf{M}^{(1)})^2$, energy $\mathcal{E}^{\mathbf{M}}_{\mathrm{triplet}^{(2)}}$ is identical to the terms in the sum defining $\mathcal{E}^{\mathbf{M}}_{\mathrm{GW}}$ for which $j' = \ell'$, and so is named for the second slice. Transport plans $\mathbf{\Pi}$ with low $\mathcal{E}^{\mathbf{M}}_{\mathrm{triplet}^{(2)}}$-energy are more likely to predict that descendants of a given spot in the first slice are close (spatially, and in feature-distance) in the second slice. Together, $\mathcal{E}^{\mathbf{M}}_{\mathrm{triplet}^{(1)}}$ and $\mathcal{E}^{\mathbf{M}}_{\mathrm{triplet}^{(2)}}$ encourage spot-wise continuity in $\mathbf{\Pi}$, without penalizing $\mathbf{\Pi}$ for spatial distortions intrinsic to tissue growth, or for change in expression characteristic of development. We refer to the sum of these terms as the *triplet* energy between triplets of points, $\mathcal{E}_{\mathrm{triplet}}$:

$$\mathcal{E}^{\mathbf{M}}_{\mathrm{triplet}}(\mathbf{\Pi}) = \mathcal{E}^{\mathbf{M}}_{\mathrm{triplet}^{(1)}}(\mathbf{\Pi}) + \mathcal{E}^{\mathbf{M}}_{\mathrm{triplet}^{(2)}}(\mathbf{\Pi}) \tag{19}$$

bioRxiv preprint doi: https://doi.org/10.1101/2024.03.05.583575; this version posted March 10, 2024. The copyright holder for this preprint (which was not certified by peer review) is the author/funder, who has granted bioRxiv a license to display the preprint in perpetuity. It is made available under aCC-BY 4.0 International license.

Combining these changes, and adding in a term of entropy regularization, the formulation [1] used by DeST-OT is:

$$\begin{aligned} \min \quad & (1-\alpha)\underbrace{\left\langle \mathbf{C}, \mathbf{\Pi} \right\rangle_F}_{\mathcal{E}_{\text{doublet}}(\mathbf{\Pi})} + \frac{\alpha}{2}\left(\mathcal{E}^{\mathbf{M}}_{\text{triplet}}(\mathbf{\Pi}) + \mathcal{E}^{\mathbf{M}}_{\text{quartet}}(\mathbf{\Pi})\right) + \gamma \text{KL}(\mathbf{\Pi}\mathbf{1}_{n_2} \parallel \mathbf{g}_1) - \epsilon \text{H}(\mathbf{\Pi}) \\ s.t. \quad & \mathbf{\Pi}^T \mathbf{1}_{n_1} = \mathbf{g}_2 = \frac{1}{n_1}\mathbf{1}_{n_2}, \quad \mathbf{g}_1 = \frac{1}{n_1}\mathbf{1}_{n_1}, \quad \mathbf{\Pi} \succcurlyeq 0 \end{aligned} \tag{20}$$

with $\mathbf{L}^{\mathbf{M}}$ defined in (16), and the notation $\mathbf{L}^{\mathbf{M}} \otimes \mathbf{\Pi}$ as defined just below (13). The choices of $\mathbf{V}^{(1)} = ((\mathbf{M}^{(1)})^2$ and $\mathbf{V}^{(2)} = ((\mathbf{M}^{(2)})^2$ as above enable us to write the sum of our triplet and quartet energies in (20) as

$$\left\langle \widetilde{\mathbf{L}}^{\mathbf{M}} \otimes \mathbf{\Pi}, \mathbf{\Pi} \right\rangle = \sum_{ij'kl'} (1 + \delta_{ik} + \delta_{j'l'}) \left(\mathbf{M}^{(1)}_{ik} - \mathbf{M}^{(2)}_{j'l'}\right)^2 \mathbf{\Pi}_{ij'} \mathbf{\Pi}_{kl'} \tag{21}$$

where δ_{ik} is 1 if and only if $i = k$ and is 0 otherwise. The 4-tensor $\widetilde{\mathbf{L}}^{\mathbf{M}}$ is defined implicitly by the above display, and the objective function used by DeST-OT can now expressed concisely as follows, though (20) is the expression we use in practice for computing gradients:

$$(1-\alpha)\left\langle \mathbf{C}, \mathbf{\Pi} \right\rangle_F + \frac{\alpha}{2}\underbrace{\left\langle \widetilde{\mathbf{L}}^{\mathbf{M}} \otimes \mathbf{\Pi}, \mathbf{\Pi} \right\rangle_F}_{\mathcal{E}^{\mathbf{M}}(\mathbf{\Pi})} + \gamma \text{KL}(\mathbf{\Pi}\mathbf{1}_{n_2} \parallel \mathbf{g}_1) - \epsilon \text{H}(\mathbf{\Pi}) \tag{22}$$

As indicated by the underbrace in (22), we define the *merged feature-spatial energy*, denoted $\mathcal{E}^{\mathbf{M}}(\mathbf{\Pi})$ to be the sum of $\mathcal{E}^{\mathbf{M}}_{\text{quartet}}(\mathbf{\Pi})$ and $\mathcal{E}^{\mathbf{M}}_{\text{triplet}}(\mathbf{\Pi})$, to have a more descriptive way of referring to the left-hand side of (21).

S1.3 Connection to energy-based models

Owing to the presence of multiple energetic conditions, including within-slice energies and matching energies, we make a connection between the optimal transport problem we have defined and an energy-based model. For some energy function $\mathcal{E}_\theta(\boldsymbol{x}) \geq 0$, and for some variable we are optimizing $\boldsymbol{x}(\triangleq \mathbf{\Pi}$ in our case), one may define a Gibbs-Boltzmann distribution $P_\theta(\boldsymbol{x})$ given by:

$$P_\theta(\boldsymbol{x}) = \frac{1}{Z_\theta} e^{-\mathcal{E}_\theta(\boldsymbol{x})},$$

where θ comes from the SRT data, $\theta = (\mathbf{C}, \mathbf{C}^{(1)}, \mathbf{C}^{(2)}, \mathbf{X}^{(1)}, \mathbf{X}^{(2)})$. The normalizing constant (or *partition function*) Z_θ is given by $Z_\theta = \int_{\boldsymbol{x}} e^{-\mathcal{E}_\theta(\boldsymbol{x})} \mathrm{d}\boldsymbol{x}$, which is generally intractable to compute. For such an energy-based model, in order to find an optimal value for $\boldsymbol{x}$, one may optimize the the log-likelihood of the model given by $\nabla_{\boldsymbol{x}} \log P_\theta(\boldsymbol{x})$, where:

$$\nabla_{\boldsymbol{x}} \log P_\theta(\boldsymbol{x}) = \nabla_{\boldsymbol{x}} \left(-\mathcal{E}_\theta(\boldsymbol{x}) - \int_{\boldsymbol{x}} e^{-\mathcal{E}_\theta(\boldsymbol{x})} d\boldsymbol{x}\right) = -\nabla_{\boldsymbol{x}} \mathcal{E}_\theta(\boldsymbol{x})$$

which holds as the partition function is clearly a constant with respect to $\boldsymbol{x}$. For the Wasserstein energy $\langle \mathbf{C}, \boldsymbol{x} \rangle$, if $\mathcal{E}_\theta(\boldsymbol{x}) = -\langle \mathbf{C}, \boldsymbol{x} \rangle_F$ represents our energy function, we are simply solving a standard optimal-transport problem. If $\mathcal{E}_\theta(\boldsymbol{x}) = -((1-\alpha)\langle \boldsymbol{C}, \boldsymbol{x} \rangle_F + \alpha \langle \mathbf{L}^{\mathbf{D}} \otimes \mathbf{\Pi}, \mathbf{\Pi} \rangle_F)$, then we are solving an FGW optimal-transport problem, and so on. While the connection to energy-functions is trivially evident, the value of introducing the connection is seen in the product-of-experts formulation of energy-based models, where one may have multiple Gibbs-Boltzmann distributions $P^{(1)}_\theta, P^{(2)}_\theta, ..., P^{(N)}_\theta$, each carrying some "expert" information, where the distribution representing the combination of the features of all of the distributions is called a product-of-experts [16], given by:

$$P_\theta(\boldsymbol{x}) = \frac{1}{Z^{(1:N)}_\theta} \prod_{i=1}^{N} P^{(i)}_\theta(\boldsymbol{x}) = \frac{1}{Z^{(1:N)}_\theta} \prod_{i=1}^{N} \frac{1}{Z^{(i)}_\theta} e^{-\mathcal{E}^{(i)}_\theta(\boldsymbol{x})} \propto_{\boldsymbol{x}} e^{-\sum_{i=1}^{N} \mathcal{E}^{(i)}_\theta(\boldsymbol{x})}$$

Where the energy-function of the final Gibbs-Boltzmann distribution is given by the *sum* of the individual energies:

$$\mathcal{E}_\theta(\boldsymbol{x}) = \mathcal{E}^{(1)}_\theta(\boldsymbol{x}) + ... + \mathcal{E}^{(i)}_\theta(\boldsymbol{x}) + ... + \mathcal{E}^{(N)}_\theta(\boldsymbol{x})$$

[1] All matrices involved are subject to a consistent data-dependent normalization by matrix norms which ensure the terms of the gradient are scaled analogously, such that $\alpha = 0.5$ roughly means both terms contribute equal weight for interpretability and robustness of the hyper-parameter defaults to different datasets.

bioRxiv preprint doi: https://doi.org/10.1101/2024.03.05.583575; this version posted March 10, 2024. The copyright holder for this preprint (which was not certified by peer review) is the author/funder, who has granted bioRxiv a license to display the preprint in perpetuity. It is made available under aCC-BY 4.0 International license.

As such, we augment the optimal-transport formulation by framing it as a product-of-experts, constructing a total-energy which we optimize with respect to $\mathbf{\Pi}$ that carries the standard Wasserstein (doublet) energy $\langle \mathbf{C}, \mathbf{\Pi} \rangle_F$, the quartet energy $\langle \mathbf{L^M} \otimes \mathbf{\Pi}, \mathbf{\Pi} \rangle_F$ defined through the merged feature-spatial matrices, and two triplet energies which are also defined from these matrices: $\mathrm{Tr}\left[\mathbf{\Pi M}^{(2)}\mathbf{\Pi}^T\right]$ and $\mathrm{Tr}\left[\mathbf{\Pi}^T\mathbf{M}^{(1)}\mathbf{\Pi}\right]$. Denoting the optimal-transport entropic-term density $P_{\mathcal{H}}$, we minimize the following product-of-experts Gibbs-Boltzmann distribution with respect to $\mathbf{\Pi}$:

$$P_\theta(\mathbf{\Pi}) = \frac{1}{Z_\theta} P_{\mathcal{H}}(\mathbf{\Pi}) \times e^{-\mathcal{E}_{\text{expression}}(\mathbf{\Pi})} \times e^{-\mathcal{E}_{\text{GW}}(\mathbf{\Pi})} \times e^{-\mathcal{E}_{\text{triplet}^{(1)}}(\mathbf{\Pi})} \times e^{-\mathcal{E}_{\text{triplet}^{(2)}}(\mathbf{\Pi})}$$

$$= \frac{1}{Z_\theta} P_{\mathcal{H}}(\mathbf{\Pi}) \times \exp\left\{ \langle \boldsymbol{C}, \mathbf{\Pi} \rangle_F + \langle \mathbf{L^M} \otimes \mathbf{\Pi}, \mathbf{\Pi} \rangle_F + \mathrm{Tr}\left[\mathbf{\Pi}^T\mathbf{M}^{(1)}\mathbf{\Pi}\right] + \mathrm{Tr}\left[\mathbf{\Pi M}^{(2)}\mathbf{\Pi}^T\right] \right\}$$

Which offers the interpretation that taking the sum of the energy terms in our optimal-transport problem is equivalent to optimizing a product of the Boltzmann-distributions over our variable $\mathbf{\Pi}$, where this product represents a product-of-experts in which each "expert" distribution refines the matrix $\mathbf{\Pi}$ to be more biologically and spatially realistic with respect to the transcriptomic and spatial geometry of the first and second slices. Alternatively viewing each term as representing pairwise motifs (Wasserstein, or *doublet* term), ternary motifs (*triplet* term), and quaternary motifs (GW, or *quartet* term), these experts capture features ranging from lower to higher order.

S1.4 On the mass-flux term, semi-relaxed OT

The semi-relaxed approach has been explicitly discussed in [41, 12, 6, 4, 32, 7]. Blondel et al. [4] formulate a version of our problem in their Definition 3 ("semi-relaxed smooth primal"), fixing their second marginal as we do, but without using entropic regularization in order to produce sparse transport plans. These authors point out that the mixed relaxed distance introduced by Benamou in [2] is a version of semi-relaxed OT, fixing the first marginal instead of a second, and using an ℓ^2-penalty in place of a KL penalty. Interestingly, this penalty is applied directly to the analogue of $\boldsymbol{\xi}$ in this setting. A recent paper of Dong et al. [12] uses semi-relaxed transport for domain adaptation, aligning imaging data of different modalities. These authors use a Wasserstein loss, fixing the first marginal and in addition constraining the total mass transported. Vincent-Cuaz et al. [41] apply the semi-relaxed framework to graph matching, fixing the first marginal, and using a GW loss function. For matching two graphs, they find that fixing the first marginal while relaxing the second better preserves the structure of the first graph under the transport plan, versus the balanced regime. These authors [41] note that semi-relaxed OT (in the absence of entropy regularization) produces sparser transport plans than vanilla optimal transport. This sparsity also motivated the earlier work of [32] on color transfer; maps that are "too" multi-valued (i.e. not sparse enough) can inappropriately mix colors being transferred and create spatial irregularities. These authors also fix the first marginal, using a sparse transport plan to assign colors from the second image to the pixel of the first, while preserving its geometry. On the theoretical side, Chizat et al. [7] introduce the notion of a semi-coupling to connect the (PDE) dynamic and static pictures of semi-relaxed OT. Lastly, we mention two studies of Frank-Wolfe [13] and Sinkhorn [14] in the semi-relaxed setting.

We now discuss some properties of the mass-flux term $\boldsymbol{\xi}$ built from $\mathbf{\Pi}$ and advantages our formulation give us. Let us start by examining the objects analogous to $\boldsymbol{\xi}$ in the fully-unbalanced case. When both marginal constraints are relaxed, both of the vectors $\boldsymbol{\xi}_1 = \mathbf{\Pi}\mathbf{1}_{n_2} - \mathbf{g}_1$ and $\boldsymbol{\xi}_2 = \mathbf{\Pi}^T\mathbf{1}_{n_1} - \mathbf{g}_2$ may be non-zero. Towards relating vectors $\boldsymbol{\xi}_1$ and $\boldsymbol{\xi}_2$, we take the inner product of these expressions with $\mathbf{1}_{n_i}$ to obtain:

$$\mathbf{1}_{n_1}^T\boldsymbol{\xi}_1 = \mathbf{1}_{n_1}^T\mathbf{\Pi}\mathbf{1}_{n_2} - \mathbf{1}_{n_1}^T\mathbf{g}_1 \quad \text{and} \quad \mathbf{1}_{n_2}^T\boldsymbol{\xi}_2 = \mathbf{1}_{n_2}^T\mathbf{\Pi}^T\mathbf{1}_{n_1} - \mathbf{1}_{n_2}\mathbf{g}_2,$$

We solve each of these for the total mass transported, namely $\mathbf{1}_{n_1}^T\mathbf{\Pi}\mathbf{1}_{n_2} \equiv \mathbf{1}_{n_2}^T\mathbf{\Pi}^T\mathbf{1}_{n_1}$. After relating the two expressions through the total mass transported and re-arranging terms, one has

$$\begin{aligned}\mathbf{1}_{n_1}^T\boldsymbol{\xi}_1 - \mathbf{1}_{n_2}^T\boldsymbol{\xi}_2 &= \mathbf{1}_{n_2}^T\mathbf{g}_2 - \mathbf{1}_{n_1}^T\mathbf{g}_1 \\ &\equiv \frac{n_2 - n_1}{n_1},\end{aligned}$$

with the last line following from our choice of normalization on the uniform measures $\mathbf{g}_1 = \frac{1}{n_1}\mathbf{1}_{n_1}$ and $\mathbf{g}_2 = \frac{1}{n_1}\mathbf{1}_{n_2}$.

In the semi-relaxed context of `DeST-OT`, $\boldsymbol{\xi} \equiv \boldsymbol{\xi}_1$, and vector $\boldsymbol{\xi}_2$ is zero, in which case, one has

$$\mathbf{1}_{n_1}^T\boldsymbol{\xi} = \frac{n_2 - n_1}{n_1}.$$

bioRxiv preprint doi: https://doi.org/10.1101/2024.03.05.583575; this version posted March 10, 2024. The copyright holder for this preprint (which was not certified by peer review) is the author/funder, who has granted bioRxiv a license to display the preprint in perpetuity. It is made available under aCC-BY 4.0 International license.

Thus, the semi-relaxed formulation allows us to describe all growth and death implicit in transport plan $\mathbf{\Pi}$ by a single vector $\boldsymbol{\xi}$, instead of a pair of vectors $(\boldsymbol{\xi}_1, \boldsymbol{\xi}_2)$ of different dimensionality. Moreover, the hard constraint of the semi-relaxed formulation makes this single vector $\boldsymbol{\xi}$ interpretable in a way that the pair $(\boldsymbol{\xi}_1, \boldsymbol{\xi}_2)$ is not. Observe that the second marginal $\mathbf{g}_2$ is fixed, with units assigning mass $\frac{1}{n_1}$ to each spot. Though the first marginal of $\mathbf{\Pi}$ is no longer fixed, $\boldsymbol{\xi}$ is by definition an additive perturbation of $\mathbf{g}_1$ which uses the same units as $\mathbf{g}_2$, also assigning mass $\frac{1}{n_1}$ to each spot. $\boldsymbol{\xi}$ also uses these units, giving expression $\mathbf{g}_1 + \boldsymbol{\xi}$ physical meaning.

Rescaled vector $\mathbf{q}_1 = n_1(\mathbf{\Pi}\mathbf{1}_{n_2})$ has in its i-th element the amount of mass transported from spot i, in units of *numbers of spots*. These numbers are fractional, as $\mathbf{\Pi}$ is implicitly estimating these values in a sense averaged over $t_2 - t_1$, with respect to a stochastic process similar to the one described in [6]. Because the rescaled target measure $n_1\mathbf{g}_2$ shares these counting-measure units, we can reasonably interpret $\mathbf{q}_1 \equiv \mathbf{q}_1(\mathbf{\Pi})$ as the vector of the *expected number of descendants* of each spot in the first slice, as predicted by $\mathbf{\Pi}$. This is clear, as we have:

$$\mathbb{E}_{\sim \mathcal{S}_1,\mathcal{S}_2}[\sum_{j\in\mathcal{S}_2} \mathbb{1}[i \to j]] = \sum_{j\in\mathcal{S}_2} \mathbb{E}_{\sim \mathcal{S}_1,\mathcal{S}_2}\left[\mathbb{1}[i \to j]\right] = \sum_{j\in\mathcal{S}_2} \mathbf{\Pi}_{i,j} \triangleq \mathbf{q}_{1,i}/n_1$$

Where multiplying by n_1 scales $\mathbf{q}_1$ to have the units of counting measure over spots. We lose this interpretation in the fully-unbalanced regime: when the target measure is not constrained to be uniform, the amount of mass transported *from* spot i may not reflect an expected number of descendants, as this value will depend on the mass of each descendant.

From the definitions of $\boldsymbol{\xi}$ and $\mathbf{q}_1$, we have

$$n_1\boldsymbol{\xi} = \mathbf{q}_1 - \mathbf{1}_{n_1},$$

and $n_1\boldsymbol{\xi}$ has the following intuitive interpretation: at spot i,

$$n_1\boldsymbol{\xi}_i = \text{Expected number of } \textbf{branches/descendants} \text{ contributed from spot i}$$

S2 Growth rate conversion

The conversion of the growth vector (mass-flux) $\boldsymbol{\xi}$ to a growth rate can be done simply. We use the growth process described in [22], where one considers the PDE modeling a transcriptomic trajectory as a density evolving in time:

$$\frac{\partial \rho}{\partial t} = -\boldsymbol{\nabla} \cdot (\rho \mathbf{F}) + \frac{\sigma^2}{2}\Delta\rho + J\rho$$

With $-\boldsymbol{\nabla} \cdot (\rho\mathbf{F})$ a continuity equation drift term, $\frac{\sigma^2}{2}\Delta\rho$ a diffusion term, and $J\rho$ a term representing growth and branching, which are all modeled using successive optimal transport steps. We convert our mass-flux term from the optimal transport to a growth using the principle of [22], where operator-splitting the branching component above from the drift-diffusion gives us a continuous-time growth update as:

$$\partial_t \rho = J\rho$$

Thus we simply need to consider the solution to the ordinary differential equation $\dot{\rho} = J\rho$ for J between times t_1, t_2:

$$\rho(t_2) = e^{J(t_2-t_1)}\rho(t_1)$$

For $\rho(t_1)$ and $\rho(t_2)$ a prior and posterior density over the spots in the first slice. By the semi-relaxed optimal transport formulation, we simply compare against a simple uniform prior density $\rho(t_1) = \frac{1}{n_1}$ which we start with. Thus one concludes the connection to a growth rate, on a per-spot basis in the first slice, is given as:

$$J = \frac{\log\left(\rho(t_2)n_1\right)}{t_2 - t_1} = \frac{\log\left(n_1\xi + 1\right)}{t_2 - t_1}$$

Which establishes a simple monotonic connection between our growth vector $\boldsymbol{\xi}$ and a proper growth rate in time. In the notation of [22], $\tau = t_2 - t_1$, and one has $J = \tau^{-1}(p_b - p_d)$, establishing a connection between the growth rate and birth-death parameters p_b, p_d, and τ. Conventionally, cell division and death are modeled as follows: each cell has an exponential clock of rate τ^{-1}. When a cell's clock rings, it dies with probability p_d, or splits into two cells with probability $p_b = 1 - p_d$. As such, our growth rates have a natural connection to the parameters of this birth-death process.

bioRxiv preprint doi: https://doi.org/10.1101/2024.03.05.583575; this version posted March 10, 2024. The copyright holder for this preprint (which was not certified by peer review) is the author/funder, who has granted bioRxiv a license to display the preprint in perpetuity. It is made available under aCC-BY 4.0 International license.

S3 Connection to the continuity equation and interpretation of growth vector

Suppose we denote $\mathbf{x}(t_k) = \mathbf{x} \sim \mu$, $\mathbf{x}(t_{k+1}) = \mathbf{y} \sim \nu$ as samples of our slices $\mathcal{S}_1$ and $\mathcal{S}_2$, and consider the pairwise-alignment problem in the equivalent Monge-formulation (the discrete analogue of μ being $\mathbf{g_1}$, and ν being $\mathbf{g_2}$). The Monge-problem is formulated as:

$$\inf_{\mathbf{T}:\mathbf{T}_{\#}\mu=\nu} \int_{\mathbb{R}^d} \|\mathbf{T}(\mathbf{x}) - \mathbf{x}\|_2 d\mu(\mathbf{x})$$

Where it can be shown that an equivalent form exists in which the transport-map can be expressed as the integral of some vector field $\mathbf{F}(\mathbf{x}, t)$, weighted by a time-dependent density $\rho(\mathbf{x}, t)$ and where the optimization is performed over a vector-field of least-norm:

$$\begin{aligned} \mathcal{W}_2^2(\mu, \nu) = \inf_{(\rho, \mathbf{F})} \quad & T \int_{\mathbb{R}^d} \int_0^T \rho(\mathbf{x}, t) \|\mathbf{F}(\mathbf{x}, t)\|_2^2 d\mathbf{x} dt \\ \text{s.t.} \quad & \rho(\cdot, t_k) = \mu \\ & \rho(\cdot, t_{k+1}) = \nu \end{aligned} \tag{23}$$

Where the vector field is optimized subject to the continuity equation: $\partial_t \rho = -\boldsymbol{\nabla} \cdot (\rho \mathbf{F}(\mathbf{x}, t))$ [39]. In the semi-relaxed case, one may either assume mass-conservation or generalize the vanilla continuity equation by assuming the presence of a source or sink term σ (i.e. $\sigma = J\rho$ for J a growth rate as defined in S2) which allows mass to be introduced into the system in the form $\partial_t \rho = -\boldsymbol{\nabla} \cdot (\rho \mathbf{F}(\mathbf{x}, t)) + J\rho$. As such, $\boldsymbol{\xi}$ represents the integral of a divergence in the case of mass-conservation (i.e. local mass-redistribution), or a spatial divergence added to a source/sink term in the case where the total mass is increased or decreased in the system. To be precise, we have that in the balanced case:

$$\boldsymbol{\xi}(\mathbf{x}) = \int_{t_1}^{t_2} \left(\frac{\partial \rho}{\partial t} \right) dt = \int_{t_1}^{t_2} -\boldsymbol{\nabla} \cdot (\rho \mathbf{F}(\mathbf{x}, t))\, dt$$

And general semi-relaxed case:

$$\boldsymbol{\xi}(\mathbf{x}) = \int_{t_1}^{t_2} \left(\frac{\partial \rho}{\partial t} \right) dt = \int_{t_1}^{t_2} (\, -\boldsymbol{\nabla} \cdot \rho \mathbf{F}(\mathbf{x}, t) + \sigma \,)\, dt$$

S3.1 Interpretation of ξ

Supposing we call the spatial volume encompassed by our tissue slice at time t, V_t. Each element of ξ, ξ_k, represents the approximate value of $\boldsymbol{\nabla} \cdot (\rho(x_k, y_k)\mathbf{F}(\mathbf{x}, t)(x_k, y_k))$ in some box $[x_k - \Delta, x_k + \Delta] \times [y_k - \Delta, y_k + \Delta]$ centered at $(x_k, y_k) \in V_t$. Generally speaking the volume V_t is assumed to be connected such that, for N_t dots at time t, the volume is $V_t = \cup_{k=1}^{N_t} [x_k - \Delta, x_k + \Delta] \times [y_k - \Delta, y_k + \Delta]$.

We assume a cell type assignment is some function from a matrix of distance-based dissimilarities and feature-values to a partition $\mathcal{P}$ of the data $\phi(D, C) = \mathcal{P}$. Where $\mathcal{P} = \{\mathcal{P}_k\}_{k=1}^K$ represents a decomposition of our dots into disjoint sets representing respective cell types. Following from this, we define the volume of a cell type k at time t, $V_{t,k}$, as $V_{t,k} = \cup_{(x_i, y_i) \in \mathcal{P}_k} [x_i - \Delta, x_i + \Delta] \times [y_i - \Delta, y_i + \Delta]$. Clearly, $V_t = \cup_{k=1}^K V_{t,k}$.

Definition 1. *Tissue-slice growth. The term* $\mathbf{1}^T \boldsymbol{\xi} = \sum_x \sum_y \int_{t_1}^{t_2} -\boldsymbol{\nabla} \cdot (\rho(x, y)\mathbf{F}(\mathbf{x}, t)(x, y))$ *represents the discretized volume integral*

$$\int_{t_1}^{t_2} \iint_{V_t} -\boldsymbol{\nabla} \cdot (\rho(x, y)\mathbf{F}(\mathbf{x}, t)(x, y))\, dV_t = -\int_{t_1}^{t_2} \oint_{\partial V_t = S_t} (\rho \mathbf{F}(\mathbf{x}, t)) \cdot \hat{n} dS_t$$

for $\Delta = 1/2$*. By the divergence theorem, one identifies the sum of the divergences in the volume* V_t *as the mass-flux across the boundary* ∂V_t*. Thus, the growth of a tissue slice contained within in a volume at time t,* V_t*, is defined as the mass-flux across this boundary integrated from* t_1 *to* t_2*.*

For a balanced optimal transport this is identically zero for any boundary as $\boldsymbol{\xi} = \mathbf{0}$:

$$\mathbf{1}_{n_1}^T \boldsymbol{\xi} = \mathbf{1}_{n_1}^T (\boldsymbol{\Pi} \mathbf{1}_{n_2} - \mathbf{g_1}) = \mathbf{1}_{n_1}^T \mathbf{0}_{n_1} = 0$$

bioRxiv preprint doi: https://doi.org/10.1101/2024.03.05.583575; this version posted March 10, 2024. The copyright holder for this preprint (which was not certified by peer review) is the author/funder, who has granted bioRxiv a license to display the preprint in perpetuity. It is made available under aCC-BY 4.0 International license.

For a semi-relaxed optimal transport with marginals that sum to the same value, such as the uniform marginals $\Pi^{\mathbf{T}}\mathbf{1}_{n_1} = \mathbf{g}_2 = \frac{1}{n_2}\mathbf{1}_{n_2}$ and marginal $\mathbf{g}_1 = \frac{1}{n_1}\mathbf{1}_{n_1}$, one may have *local* growth or death as $\boldsymbol{\xi}$ is not necessarily the zero-vector, but globally mass is conserved:

$$\mathbf{1}_{n_1}^T\boldsymbol{\xi} = \left(\Pi^{\mathbf{T}}\mathbf{1}_{n_1}\right)^{\mathbf{T}}\mathbf{1}_{n_2} - \mathbf{1}_{n_1}^T\mathbf{g}_1 = \mathbf{g}_2^T\mathbf{1}_{n_2} - \mathbf{1}_{n_2}^T\mathbf{g}_1 = 0$$

In the case of a semi-relaxed optimal transport, if we allow for this source or sink term where

$$\mathbf{1}^T\boldsymbol{\xi} = \int_{t_1}^{t_2} \oint_{\partial V_t = S_t} \left(-\rho\mathbf{F}(\mathbf{x},t) + \sigma\right)\cdot \hat{n}\, dS_t$$

we generalize the above definition by asserting global mass gain or loss is possible. In particular, we can set the unbalanced marginals to reflect the normalized change in volume (or change in mass) as:

$$\mathbf{1}_{n_1}^T\boldsymbol{\xi} = \mathbf{g}_2^T\mathbf{1}_{n_2} - \mathbf{1}_{n_1}^T\mathbf{g}_1 = \eta \tag{24}$$

Where $\eta \in \mathbb{R}$ could be chosen to reflect something known–such as a total change of mass or volume between the two slices. We also note that the interpretation of $\boldsymbol{\xi}$ can also be used to measure local growth of a tissue within some fixed boundary, as described in the corollary below.

Corollary 1 (Cell type growth)**.** *Given a partition $\mathcal{P}$ of tissue into separate cell types, and defining the vector $\mathbf{z}_p$ by*

$$(\mathbf{z}_p)_k = \begin{cases} 1 \text{ if } (x_k, y_k) \in \mathcal{P}_p \\ 0 \text{ otherwise} \end{cases}$$

The term $\mathbf{z}_p^T\xi$ represents the volume integral

$$\int_{t_1}^{t_2}\iint_{V_{t,p}} -\boldsymbol{\nabla}\cdot\left(\rho(x,y)\mathbf{F}(\mathbf{x},t)(x,y)\right)dV_{t,p} = -\int_{t_1}^{t_2}\oint_{\partial V_{t,p} = S_{t,p}} \left(\rho\mathbf{F}(\mathbf{x},t)\right)\cdot\hat{n}\, dS_{t,p}$$

and represents the mass-flux out of the boundary of cell type p, $\partial V_{t,p}$, integrated from t_1 to t_2.

S3.2 Defining an Unbalanced Optimal Transport problem to match an a priori known total mass-flux

We seek a balancing condition which fixes the mass-flux η from our optimal transport 24 to some interpretable and meaningful value. By our previous decomposition of the volume of a bar-coded grid of spots into boxes of equal volume Δ^2, or analogously a small hexagonal volume for Visium, and our assumption that the mass-density is constant for each spot, we have that the normalized mass-flux above must equate to:

$$= \frac{\int_{\mathcal{S}_2}\rho_M(\mathbf{x}_2)dV(\mathbf{x}_2) - \int_{\mathcal{S}_1}\rho_M(\mathbf{x}_1)dV(\mathbf{x}_1)}{\int_{\mathcal{S}_1}\rho_M(\mathbf{x}_1)dV(\mathbf{x}_1)} = \frac{\int_{\mathcal{S}_2}dV(\mathbf{x}_2) - \int_{\mathcal{S}_1}dV(\mathbf{x}_1)}{\int_{\mathcal{S}_1}dV(\mathbf{x}_1)} \triangleq \frac{1}{|\mathbf{vol}\,(\mathcal{S}_1)\,|}\int_{t_1}^{t_2}\left(\frac{\partial\mathbf{vol}S}{\partial t}\right)dt,$$

as we assume that the mass-density $\rho_M(\mathbf{x}) = C$ is constant for any spot on the grid.

For Δ^2 a per-spot volume, we see that the volume-normalization lets this technology-specific factor cancel and the sum of the growth $\boldsymbol{\xi}$ is related to the change-in-spots directly as

$$\mathbf{1}_{n_1}^T\boldsymbol{\xi} = \frac{|\mathbf{vol}\,(\mathcal{S}_2)\,| - |\mathbf{vol}\,(\mathcal{S}_1)\,|}{|\mathbf{vol}\,(\mathcal{S}_1)\,|} = \frac{\Delta^2(n_2 - n_1)}{\Delta^2 n_1} = \frac{n_2 - n_1}{n_1}$$

Which requires that the unbalanced condition have $\mathbf{g}_2 = \frac{1}{n_1}\mathbf{1}_{n_2}$ as the constraint measure. This makes sense, as $\mathbf{1}_{n_1}^T\mathbf{g}_1 = \mathbf{1}_{n_1}^T\mathbf{1}_{n_1}\frac{1}{n_1} = 1$ and $\mathbf{1}_{n_2}^T\mathbf{g}_2 = \mathbf{1}_{n_2}^T\mathbf{1}_{n_2}\frac{1}{n_1} = \frac{n_2}{n_1}$, which has the interpretation that the density in the first slice sums to 1, and the density of the second is proportional to the ratio of the number of spots of the second slice to the first. With $\frac{n_2}{n_1} > 1$ implying mass is generated as the second volume exceeds the first, $\frac{n_2}{n_1} < 1$ implying mass is lost as the second volume is smaller than the first, and $\frac{n_2}{n_1} = 1$ implying mass-conservation.

bioRxiv preprint doi: https://doi.org/10.1101/2024.03.05.583575; this version posted March 10, 2024. The copyright holder for this preprint (which was not certified by peer review) is the author/funder, who has granted bioRxiv a license to display the preprint in perpetuity. It is made available under aCC-BY 4.0 International license.

S4 Semi-relaxed Sinkhorn

Calculating the gradient with respect to $\mathbf{\Pi}$

In order to run Sinkhorn, we require an initial condition on $\mathbf{\Pi}$ and an analytical expression for the gradient of our primal objective with respect to $\mathbf{\Pi}$. In particular, the following represents a primal feasible $\mathbf{\Pi}$:

$$\mathbf{\Pi}_0 = \frac{1}{n_1 n_2}\mathbf{1}_{n_1}\mathbf{1}_{n_2}^T \succcurlyeq 0,$$
$$\mathbf{\Pi}_0^T\mathbf{1}_{n_1} = \frac{1}{n_1 n_2}\mathbf{1}_{n_2}\mathbf{1}_{n_1}^T\mathbf{1}_{n_1} = \mathbf{g}_2$$

Recall that the energy function $\mathcal{E} : \mathbf{\Pi} \mapsto \mathcal{E}(\mathbf{\Pi})$ of interest in our current formulation is

$$\mathcal{E}(\mathbf{\Pi}) = \underbrace{(1-\alpha)\Big\langle \mathbf{C}, \mathbf{\Pi}\Big\rangle_F}_{\mathcal{E}_{\text{expression}}(\mathbf{\Pi})} + \underbrace{\frac{\alpha(1-\beta)}{2}\Big\langle \mathbf{L}^{\mathbf{\Psi}} \otimes \mathbf{\Pi}, \mathbf{\Pi}\Big\rangle_F}_{\mathcal{E}^{\mathbf{\Psi}}_{\text{GW}}(\mathbf{\Pi})} + \underbrace{\frac{\alpha\beta}{2}\operatorname{Tr}\Big[\mathbf{\Pi}\mathbf{\Psi}^{(2)}\mathbf{\Pi}^T\Big]}_{\mathcal{E}^{\mathbf{\Psi}^{(2)}}_{\text{triplet}^{(1)}}(\mathbf{\Pi})} + \underbrace{\frac{\alpha\beta}{2}\operatorname{Tr}\Big[\mathbf{\Pi}^T\mathbf{\Psi}^{(1)}\mathbf{\Pi}\Big]}_{\mathcal{E}^{\mathbf{\Psi}^{(1)}}_{\text{triplet}^{(2)}}(\mathbf{\Pi})} \tag{25}$$
$$+ \gamma \mathrm{KL}(\mathbf{\Pi}\mathbf{1}_{n_2} \parallel \mathbf{g}_1) - \epsilon \mathrm{H}(\mathbf{\Pi})$$

We calculate the gradient of f as follows. The gradient of the expression term $\mathcal{E}_{\text{expression}}(\mathbf{\Pi})$ is straightforward to compute:

$$\nabla_{\mathbf{\Pi}}\mathcal{E}_{\text{expression}}(\mathbf{\Pi}) = (1-\alpha)\mathbf{C}$$

Next, we consider the gradient of the distance-regularization term $\mathcal{E}_{\text{row}}$ corresponding to the rows of $\mathbf{\Pi}$:

$$\begin{aligned} D\mathcal{E}^{\mathbf{\Psi}^{(2)}}_{\text{triplet}^{(1)}}(\mathbf{\Pi}) \circ [\mathbf{V}] &= \frac{\alpha\beta}{2}\operatorname{Tr}\Big[D\left(\mathbf{\Pi}\mathbf{\Psi}^{(2)}\mathbf{\Pi}^T\right) \circ [\mathbf{V}]\Big] \\ &= \frac{\alpha\beta}{2}\operatorname{Tr}\Big[\mathbf{V}\mathbf{\Psi}^{(2)}\mathbf{\Pi}^T + \mathbf{\Pi}\mathbf{\Psi}^{(2)}\mathbf{V}^T\Big] \\ &= \frac{\alpha\beta}{2}\Big\langle \mathbf{\Pi}\left(\mathbf{\Psi}^{(2)} + (\mathbf{\Psi}^{(2)})^{\mathrm{T}}\right), \mathbf{V}\Big\rangle_F \\ &= \alpha\beta\Big\langle \mathbf{\Pi}\mathbf{\Psi}^{(2)}, \mathbf{V}\Big\rangle_F \end{aligned}$$

So, we have that:

$$\nabla_{\mathbf{\Pi}}\mathcal{E}^{\mathbf{\Psi}^{(2)}}_{\text{triplet}^{(1)}}(\mathbf{\Pi}) = \alpha\beta\mathbf{\Pi}\mathbf{\Psi}^{(2)}$$

The gradient of the distance regularization term corresponding to the columns of $\mathbf{\Pi}$ is similarly given as:

$$\nabla_{\mathbf{\Pi}}\mathcal{E}^{\mathbf{\Psi}^{(1)}}_{\text{triplet}^{(2)}}(\mathbf{\Pi}) = \alpha\beta\mathbf{\Psi}^{(1)}\mathbf{\Pi}$$

We now compute the gradient of f_{KL}. Let $\text{proj}_{\Delta_{n_1-1}} = \left(\boldsymbol{I}_{n_1} - \frac{1}{n_1}\mathbf{1}_{n_1}\mathbf{1}_{n_1}^T\right)$ denote the projection onto the probability-simplex Δ_{n_1-1}

$$\begin{aligned} Df_{\text{KL}}(\mathbf{\Pi}) \circ [\mathbf{V}] &= \gamma D\mathrm{KL}(\mathbf{\Pi}\mathbf{1}_{n_2} \| \mathbf{g}_1) \circ [\mathbf{V}] \\ &= \gamma\left(\text{proj}_{\Delta_{n_1-1}}(\log(\mathbf{\Pi}\mathbf{1}_{n_2}) - \log(\mathbf{g}_1))\right)^T \mathbf{V}\mathbf{1}_{n_2} \\ &= \gamma\operatorname{Tr}\left(\mathbf{1}_{n_2}\left(\text{proj}_{\Delta_{n_1-1}}(\log(\mathbf{\Pi}\mathbf{1}_{n_2}) - \log(\mathbf{g}_1))\right)^T \mathbf{V}\right) \end{aligned}$$

With, and the $\log$ applied element-wise above. So:

$$\nabla_{\mathbf{\Pi}} f_{\text{KL}}(\mathbf{\Pi}) = \gamma\left(\boldsymbol{I}_{n_1} - \frac{1}{n_1}\mathbf{1}_{n_1}\mathbf{1}_{n_1}^T\right)(\log(\mathbf{\Pi}\mathbf{1}_{n_2}) - \log(\mathbf{g}_1))\mathbf{1}_{n_2}^T$$

bioRxiv preprint doi: https://doi.org/10.1101/2024.03.05.583575; this version posted March 10, 2024. The copyright holder for this preprint (which was not certified by peer review) is the author/funder, who has granted bioRxiv a license to display the preprint in perpetuity. It is made available under aCC-BY 4.0 International license.

Lastly, we compute the semi-relaxed gradient for $f_{\mathrm{FGW}}(\mathbf{\Pi})$:

$$\left\langle \mathbf{L}^{\mathbf{\Psi}} \otimes \mathbf{\Pi}, \mathbf{\Pi} \right\rangle_F = \sum_{i,k,j',l'} \left(\mathbf{\Psi}^{(1)}_{ik} - \mathbf{\Psi}^{(2)}_{j'l'} \right)^2 \mathbf{\Pi}_{ij'} \mathbf{\Pi}_{kl'}$$

$$= \sum_{i,k,j',l'} \left(\mathbf{\Psi}^{(1)}_{ik} \right)^2 \mathbf{\Pi}_{ij'} \mathbf{\Pi}_{kl'} - 2 \sum_{i,k,j',l'} \mathbf{\Psi}^{(1)}_{ik} \mathbf{\Psi}^{(2)}_{j'l'} \mathbf{\Pi}_{ij'} \mathbf{\Pi}_{kl'} + \sum_{j',l'} \left(\mathbf{\Psi}^{(2)}_{j'l'} \right)^2 \left(\sum_i \mathbf{\Pi}_{ij'} \left(\sum_k \mathbf{\Pi}_{kl'} \right) \right)$$

Owing to our semi-relaxed marginalization constraints, it holds that $\sum_k \mathbf{\Pi}_{kl'} = \frac{1}{n_1}$ and $\sum_i \mathbf{\Pi}_{ij'} = \frac{1}{n_1}$, such that the rightmost term is a constant and disappears when we take the gradient with respect to $\mathbf{\Pi}$. As such, the above is proportional to:

$$\propto \sum_{i,k,j',l'} \left(\mathbf{\Psi}^{(1)}_{ik} \right)^2 \mathbf{\Pi}_{ij'} \mathbf{\Pi}_{kl'} - 2 \sum_{i,k,j',l'} \mathbf{\Psi}^{(1)}_{ik} \mathbf{\Psi}^{(2)}_{j'l'} \mathbf{\Pi}_{ij'} \mathbf{\Pi}_{kl'}$$

Converting this to matrix form, we have:

$$= \mathbf{1}_{\mathbf{n_2}}^{\mathbf{T}} \mathbf{\Pi}^{\mathbf{T}} \left(\mathbf{\Psi}^{(1)} \right)^2 \mathbf{\Pi} \mathbf{1}_{\mathbf{n_2}} - 2 \operatorname{Tr} \left[\mathbf{\Pi}^{\mathbf{T}} \mathbf{\Psi}^{(1)} \mathbf{\Pi} \mathbf{\Psi}^{(2)} \right]$$

We consider each term independently. For the rightmost we consider the Fréchet derivative in the direction $\mathbf{V}$:

$$D \left(-2 \operatorname{Tr} \left[\mathbf{\Pi}^{\mathbf{T}} \mathbf{\Psi}^{(1)} \mathbf{\Pi} \mathbf{\Psi}^{(2)} \right] \right) \circ [\mathbf{V}] = -2 \operatorname{Tr} \left[\mathbf{V}^{\mathbf{T}} \mathbf{\Psi}^{(1)} \mathbf{\Pi} \mathbf{\Psi}^{(2)} + \mathbf{\Pi}^{\mathbf{T}} \mathbf{\Psi}^{(1)} \mathbf{V} \mathbf{\Psi}^{(2)} \right] = -4 \langle \mathbf{V}, \mathbf{\Psi}^{(1)} \mathbf{\Pi} \mathbf{\Psi}^{(2)} \rangle_F$$

For the leftmost term, we have:

$$D \left(\mathbf{1}_{\mathbf{n_2}}^{\mathbf{T}} \mathbf{\Pi}^{\mathbf{T}} \left(\mathbf{\Psi}^{(1)} \right)^2 \mathbf{\Pi} \mathbf{1}_{\mathbf{n_2}} \right) \circ [\mathbf{V}] = \mathbf{1}_{\mathbf{n_2}}^{\mathbf{T}} \mathbf{\Pi}^{\mathbf{T}} \left(\mathbf{\Psi}^{(1)} \right)^2 \mathbf{V} \mathbf{1}_{\mathbf{n_2}} + \mathbf{1}_{\mathbf{n_2}}^{\mathbf{T}} \mathbf{V}^{\mathbf{T}} \left(\mathbf{\Psi}^{(1)} \right)^2 \mathbf{\Pi} \mathbf{1}_{\mathbf{n_2}}$$

$$= \operatorname{Tr} \left[\mathbf{V}^{\mathbf{T}} \left(\mathbf{\Psi}^{(1)} \right)^2 \mathbf{\Pi} \mathbf{1}_{\mathbf{n_2}} \mathbf{1}_{\mathbf{n_2}}^{\mathbf{T}} \right] + \operatorname{Tr} \left[\mathbf{1}_{\mathbf{n_2}} \mathbf{1}_{\mathbf{n_2}}^{\mathbf{T}} \mathbf{\Pi}^{\mathbf{T}} \left(\mathbf{\Psi}^{(1)} \right)^2 \mathbf{V} \right] = 2 \langle \mathbf{V}, \left(\mathbf{\Psi}^{(1)} \right)^2 \mathbf{\Pi} \mathbf{1}_{\mathbf{n_2}} \mathbf{1}_{\mathbf{n_2}}^{\mathbf{T}} \rangle_F$$

We can therefore identify the final gradient of our semi-relaxed GW term as:

$$\nabla_{\mathbf{\Pi}} f_{\mathrm{FGW}}(\mathbf{\Pi}) = \frac{\alpha(1-\beta)}{2} \left(2 \left(\mathbf{\Psi}^{(1)} \right)^2 \mathbf{\Pi} \mathbf{1}_{\mathbf{n_2}} \mathbf{1}_{\mathbf{n_2}}^{\mathbf{T}} - 4 \mathbf{\Psi}^{(1)} \mathbf{\Pi} \mathbf{\Psi}^{(2)} \right) = \alpha(1-\beta) \left(\left(\mathbf{\Psi}^{(1)} \right)^2 \mathbf{\Pi} \mathbf{1}_{\mathbf{n_2}} \mathbf{1}_{\mathbf{n_2}}^{\mathbf{T}} - 2 \mathbf{\Psi}^{(1)} \mathbf{\Pi} \mathbf{\Psi}^{(2)} \right)$$

With the squaring operation applied element-wise through $\mathbf{\Psi}^{(1)}$.

S4.1 Dual formulation of semi-relaxed Sinkhorn

For our optimal-transport problem, we have both a primal objective $\mathcal{E}(\mathbf{\Pi})$ we seek to minimize, as well as a single constraint that $\mathbf{\Pi}^T \mathbf{1}_{n_1} = \mathbf{g}_2$ which restricts the set of primal-feasible $\mathbf{\Pi}$. The Karush-Kuhn-Tucker (KKT) conditions introduce a set of dual variables for each constraint, and a Lagrangian which lower bounds the primal objective. The conditions, which involve first order constraints, dual constraints, and complementary slackness constraints, offer a set of conditions which must be satisfied by an optimal solution to the primal problem. As such, we introduce the dual-variable $\boldsymbol{\mu}$, and establish the Lagrangian $\mathcal{L}$ to maximize as a lower-bound to the primal objective:

$$\mathcal{L}(\mathbf{\Pi}, \boldsymbol{\mu}) = (1-\alpha) \left\langle \mathbf{C}, \mathbf{\Pi} \right\rangle_F + \frac{\alpha}{2} \left\{ (1-\beta) \left\langle \mathbf{L}^{\mathbf{\Psi}} \otimes \mathbf{\Pi}, \mathbf{\Pi} \right\rangle_F + \beta \left(\operatorname{Tr} \left[\mathbf{\Pi} \mathbf{\Psi}^{(2)} \mathbf{\Pi}^T \right] + \operatorname{Tr} \left[\mathbf{\Pi}^T \mathbf{\Psi}^{(1)} \mathbf{\Pi} \right] \right) \right\}$$
$$+ \gamma \mathrm{KL}(\mathbf{\Pi} \mathbf{1}_n || \mathbf{g}_1) + -\epsilon H(\mathbf{\Pi}) + \boldsymbol{\mu}^T \left(\mathbf{\Pi}^T \mathbf{1}_n - \mathbf{g}_2 \right) \quad (26)$$

Collecting the non-entropic terms in the gradient of the above expression, we define the matrix $\mathbf{C}^*$ to be:

$$\mathbf{C}^* \triangleq (1-\alpha) \mathbf{C} + \alpha \left(\mathbf{\Psi}^{(1)} \mathbf{\Pi} + \mathbf{\Pi} \mathbf{\Psi}^{(2)} \right) + \alpha(1-\beta) \left(\left(\mathbf{\Psi}^{(1)} \right)^2 \mathbf{\Pi} \mathbf{1}_{\mathbf{n_2}} \mathbf{1}_{\mathbf{n_2}}^{\mathbf{T}} - 2 \mathbf{\Psi}^{(1)} \mathbf{\Pi} \mathbf{\Psi}^{(2)} \right)$$

The KKT conditions imply the following first-order condition holds when we consider the gradient of our Lagrangian with respect to the variable being optimized, $\mathbf{\Pi}$:

$$\nabla_{\mathbf{\Pi}} \mathcal{L}(\mathbf{\Pi}, \boldsymbol{\mu}) = \mathbf{C}^* + \gamma \Big(\log(\mathbf{\Pi} \mathbf{1}_n) - \log(\mathbf{g}_1) \Big) \mathbf{1}_n^T + \epsilon \log \mathbf{\Pi} + \mathbf{1}_{n'} \boldsymbol{\mu}^T = \mathbf{0} \quad (27)$$

bioRxiv preprint doi: https://doi.org/10.1101/2024.03.05.583575; this version posted March 10, 2024. The copyright holder for this preprint (which was not certified by peer review) is the author/funder, who has granted bioRxiv a license to display the preprint in perpetuity. It is made available under aCC-BY 4.0 International license.

We also have the following primal constraint for our singular equality condition on the second marginal:

$$\mathbf{\Pi}^T \mathbf{1}_{n'} - \mathbf{g}_2 = \mathbf{0} \tag{28}$$

The dual constraints, which are implicitly accounted for in the entropy term of Sinkhorn which lifts the matrix $\mathbf{\Pi}$ through exponentiation such that $\mathbf{\Pi} \succcurlyeq \mathbf{0}$ always holds, for technical correctness include a dual variable $\mathbf{\Omega} \succcurlyeq \mathbf{0}$ and a term in the Lagrangian of the form $-\langle \Omega, \mathbf{\Pi} \rangle_F$. The two dual constraints would formally be given as:

$$\boldsymbol{\mu} \in \mathbb{R}^n \tag{29}$$

$$\mathbf{\Omega} \succcurlyeq \mathbf{0} \tag{30}$$

And a complementary slackness condition enforced elementwise through the matrix $\mathbf{\Pi}$, where one either has the active constraint where $\mathbf{\Pi}_{ij} > 0$ and $\mathbf{\Omega}_{ij} = 0$, or a slack, or inactive, constraint with $\mathbf{\Omega}_{ij} > 0$ for the (omitted) component of the Lagrangian given as $-\langle \mathbf{\Omega}, \mathbf{\Pi} \rangle_F$. Thus the slackness condition would be written as:

$$\mathbf{\Omega} \odot \mathbf{\Pi} = \mathbf{0} \tag{31}$$

Looking more closely at the first-order conditions, one finds:

$$(\mathbf{C}^*)_{i,j} + \gamma \Big(\log(\mathbf{\Pi}_{i,\cdot}^T \mathbf{1}_n) - \log(\mathbf{g}_{1,i}) \Big) + \epsilon \log \mathbf{\Pi}_{i,j} + \boldsymbol{\mu}_j = \mathbf{0}$$

Implying a similar solution-form to Sinkhorn involving an exponentiation with the Gibbs kernel, wherein one has $\mathbf{K}_{i,j} = e^{-\epsilon^{-1} \mathbf{C}^*_{i,j}}$ and:

$$\mathbf{\Pi}_{i,j} = e^{-\frac{\gamma}{\gamma+\epsilon} \log \left(\frac{\mathbf{\Pi}_{i,\cdot}^T \mathbf{1}_n}{\mathbf{g}_{1,i}} \right)^{(\gamma+\epsilon)/\epsilon}} \mathbf{K}_{i,j} e^{-\epsilon^{-1} \boldsymbol{\mu}_j}$$

From Sinkhorn, we know there exist unique vectors $\mathbf{u}$, $\mathbf{v}$ such that $\mathbf{\Pi} = \mathrm{diag}\,(\mathbf{u}) \mathbf{K}\, \mathrm{diag}\,(\mathbf{v})$ and see:

$$\mathbf{\Pi} = \mathrm{diag}\left(e^{\frac{-\gamma}{\epsilon} \log (\mathbf{\Pi} \mathbf{1_n} / \mathbf{g_1})} \right) \mathbf{K}\, \mathrm{diag}\left(e^{-\epsilon^{-1} \boldsymbol{\mu}} \right) \triangleq \mathrm{diag}\,(\mathbf{u}) \mathbf{K}\, \mathrm{diag}\,(\mathbf{v})$$

We consider what the update rule should be, given this identification, by establishing:

$$\mathbf{u} = e^{\frac{-\gamma}{\epsilon} \log (\mathbf{\Pi} \mathbf{1_n} / \mathbf{g_1})} = \left(\frac{\mathbf{\Pi} \mathbf{1_n}}{\mathbf{g_1}} \right)^{-\gamma/\epsilon} = \left(\frac{\mathrm{diag}\,(\mathbf{u}) \mathbf{K}\, \mathrm{diag}\,(\mathbf{v}) \mathbf{1_n}}{\mathbf{g_1}} \right)^{-\gamma/\epsilon}$$

$$= \left(\frac{\mathbf{u} \odot \mathbf{K} \mathbf{v}}{\mathbf{g_1}} \right)^{-\gamma/\epsilon} = \mathbf{u}^{-\gamma/\epsilon} \odot \left(\frac{\mathbf{K} \mathbf{v}}{\mathbf{g_1}} \right)^{-\gamma/\epsilon}$$

Noting the Hadamard multiplication, this directly implies an update rule for $\mathbf{u}$ in the form:

$$\mathbf{u} = \left(\left(\frac{\mathbf{K} \mathbf{v}}{\mathbf{g_1}} \right)^{-\gamma/\epsilon} \right)^{\frac{\epsilon}{\gamma+\epsilon}} = \left(\frac{\mathbf{g_1}}{\mathbf{K} \mathbf{v}} \right)^{\frac{\gamma}{\gamma+\epsilon}}$$

This may analogously be identified from the general form of unbalanced optimal transport, wherein one uses an anisotropic proximal step weighting two divergences on the marginals $\mathbf{g_1}$. In particular, for a generalized optimal transport problem of the form:

$$\inf_{\mathbf{\Pi}} \langle \mathbf{C}, \mathbf{\Pi} \rangle_F + \lambda_1 \mathrm{D}_{\varphi_1}(\mathbf{\Pi} \mathbf{1}_{n_2} | \mathbf{g}_1) + \lambda_2 \mathrm{D}_{\varphi_2}(\mathbf{\Pi}^T \mathbf{1}_{n_1} | \mathbf{g}_2) + \epsilon H(\mathbf{\Pi})$$

for φ_1, φ_2 convex, positive lower-semi-continuous divergences. For these, one takes a proximal step for each marginal where $\mathbf{u}$, $\mathbf{v}$ are updated as:

$$\mathbf{u} = \mathrm{argmin}_{\mathbf{u}'}\, \mathrm{KL}(\mathbf{u}' || \mathbf{u}) + \frac{\lambda_1}{\epsilon} \mathrm{D}_{\varphi_1}(\mathbf{u}' || \mathbf{g}_1)$$

$$\mathbf{v} = \mathrm{argmin}_{\mathbf{v}'}\, \mathrm{KL}(\mathbf{v}' || \mathbf{v}) + \frac{\lambda_2}{\epsilon} \mathrm{D}_{\varphi_2}(\mathbf{v}' || \mathbf{g}_2)$$

bioRxiv preprint doi: https://doi.org/10.1101/2024.03.05.583575; this version posted March 10, 2024. The copyright holder for this preprint (which was not certified by peer review) is the author/funder, who has granted bioRxiv a license to display the preprint in perpetuity. It is made available under aCC-BY 4.0 International license.

This has the following closed-form updates for unbalanced Sinkhorn where we consider the case that $\mathrm{D}_{\varphi_1}, \mathrm{D}_{\varphi_2}$ are taken to be KL-divergences:

$$\mathbf{u}^{(l+1)} = \left(\frac{\mathbf{g}_1}{\mathbf{K}\mathbf{v}^{(l)}}\right)^{\frac{\lambda_1}{\lambda_1+\epsilon}}$$

$$\mathbf{v}^{(l+1)} = \left(\frac{\mathbf{g}_2}{\mathbf{K}^T\mathbf{u}^{(l+1)}}\right)^{\frac{\lambda_2}{\lambda_2+\epsilon}}$$

While we demonstrated that the semi-relaxed update follows directly from the KKT conditions, one can easily recover the semi-relaxed update in the limit of $\lambda_2 \to \infty$ in the unbalanced case. The marginal update then becomes $\mathbf{v}^{(l+1)} = \lim_{\lambda_2\to\infty}\left(\frac{\mathbf{g}_2}{\mathbf{K}^T\mathbf{u}^{(l+1)}}\right)^{\frac{\lambda_2}{\lambda_2+\epsilon}} = \frac{\mathbf{g}_2}{\mathbf{K}^T\mathbf{u}^{(l+1)}}$, where one recovers the marginal constraint on $\mathbf{g}_2$ and our semi-relaxed update rule.

S4.2 Converting semi-relaxed Sinkhorn to log-domain for stability

Consider a general form of entropy-regularized, fully unbalanced Wasserstein optimal transport: given measures $\mathbf{g}_1 \in \mathbb{R}_+^{n_1}$, $\mathbf{g}_2 \in \mathbb{R}_+^{n_2}$, and given a candidate transport plan $\mathbf{\Pi} \in \mathbb{R}_+^{n_1\times n_2}$, let the functional $\mathbf{\Pi} \mapsto \mathcal{P}_\epsilon(\mathbf{\Pi})$ be given by

$$\mathcal{P}_\epsilon(\mathbf{\Pi}) = \langle \mathbf{C}, \mathbf{\Pi}\rangle_F + \mathrm{D}_{\varphi_1}(\mathbf{\Pi}\mathbf{1}_{n_2}|\mathbf{g}_1) + \mathrm{D}_{\varphi_1}(\mathbf{\Pi}^T\mathbf{1}_{n_1}|\mathbf{g}_2) + \epsilon\mathrm{H}(\mathbf{\Pi}) \tag{32}$$

where φ_1, φ_2 are convex, positive lower-semi-continuous, and with $\varphi_i(1) = 0$. The terms D_{φ_i} are the associated φ-divergences. Depending on the choice of the φ_i, one can recover either the fully unbalanced constraints of moscot, or the semi-relaxed setting of `DeST-OT`. Specifically, `moscot` corresponds to taking $\varphi_1 = \varphi_2 \equiv p \mapsto p\log p - p + 1$, in which case both φ-divergences are the KL divergence. On the other hand, `DeST-OT` corresponds to taking $\varphi_1 \equiv p \mapsto p\log p - p + 1$, while the single hard constraint corresponds to choosing $\varphi_2 = \iota_{\{1\}}$, where

$$\iota_{\{1\}}(p) = \begin{cases} 0 & p = 1 \\ +\infty & \text{otherwise.} \end{cases}$$

Thus, (32) corresponds to a version of the `moscot` objective (3) with the GW term $\langle \mathbf{L}\otimes\mathbf{\Pi}, \mathbf{\Pi}\rangle$ omitted, or a version of the `DeST-OT` objective (22) without our modified GW term $\langle \widetilde{\mathbf{L}}^{\mathbf{M}} \otimes \mathbf{\Pi}, \mathbf{\Pi}\rangle$. In both cases, the hyperparameters attached to different terms have been suppressed.

We define $\mathrm{OT}_\epsilon(\mathbf{g}_1, \mathbf{g}_2) = \inf_{\mathbf{\Pi}\in\mathbb{R}_+^{n_1\times n_2}} \mathcal{P}_\epsilon(\mathbf{\Pi})$, and we appeal to the dual formulation of (32), which we state as a proposition.

Proposition 1 ([36], Proposition 2)**.** *One has*

$$\mathrm{OT}_\epsilon(\mathbf{g}_1, \mathbf{g}_2) = \sup_{\mathbf{f}\in\mathbb{R}_+^{n_1}, \mathbf{g}\in\mathbb{R}_+^{n_2}} \mathcal{D}_\epsilon(\mathbf{f}, \mathbf{g}),$$

where for such $\mathbf{f} \in \mathbb{R}_+^{n_1}, \mathbf{g} \in \mathbb{R}_+^{n_2}$, *we define the dual objective to* $\mathcal{P}_\epsilon$ (32)

$$\mathcal{D}_\epsilon(\mathbf{f}, \boldsymbol{\mu}) = -\langle \varphi_1^*(-\mathbf{f}), \mathbf{g}_1\rangle_F - \langle \varphi_2^*(-\boldsymbol{\mu}), \mathbf{g}_2\rangle_F - \epsilon e^{\mathbf{f}/\epsilon} \odot e^{-\mathbf{C}/\epsilon} \odot e^{\boldsymbol{\mu}/\epsilon},$$

where $(\varphi_1^*, \varphi_2^*)$ *are the Legendre transforms of* (φ_1, φ_2), *namely* $\varphi_i^*(q) = \sup_{p\geq 0} pq - \varphi_i(p)$

The optimal primal plan $\mathbf{\Pi}^\star$ is obtained from the optimal pair $(\mathbf{f}^\star, \boldsymbol{\mu}^\star)$ of dual variables as

$$\mathbf{\Pi}^\star = \mathrm{diag}(e^{\mathbf{f}^\star/\epsilon})e^{-\mathbf{C}/\epsilon}\mathrm{diag}(e^{\boldsymbol{\mu}^\star/\epsilon}) = \mathrm{diag}\,(\mathbf{u})e^{-\mathbf{C}/\epsilon}\,\mathrm{diag}\,(\mathbf{v})$$

Thus, connecting the dual variables of the generalized Sinkhorn objective for arbitrary φ-divergences to our primal variables $\mathbf{u}$, $\mathbf{v}$, we are able to express closed-form updates for the dual variables instead. The Sinkhorn iterations for the semi-relaxed case, in terms of the dual variables $\mathbf{f}, \mathbf{g}$ are given as:

$$\mathbf{f}^{(l+1)} = \epsilon\log\mathbf{u}^{(\mathbf{l})} = \epsilon\log\left(\frac{\mathbf{g}_1}{\mathbf{K}\mathbf{v}}\right)^{\frac{\gamma}{\gamma+\epsilon}} = \frac{\gamma}{\gamma+\epsilon}\left(\epsilon\log\mathbf{g}_1 - \epsilon\log\left(\mathbf{K}e^{\boldsymbol{\mu}^{(l)}/\epsilon}\right)\right) \tag{33}$$

bioRxiv preprint doi: https://doi.org/10.1101/2024.03.05.583575; this version posted March 10, 2024. The copyright holder for this preprint (which was not certified by peer review) is the author/funder, who has granted bioRxiv a license to display the preprint in perpetuity. It is made available under aCC-BY 4.0 International license.

$$\boldsymbol{\mu}^{(l+1)} = \epsilon \log \mathbf{g}_2 - \epsilon \log \left(\mathbf{K}^T e^{\mathbf{f}^{(l+1)}/\epsilon} \right) \tag{34}$$

Owing to the presence of the exponential of each dual variable on the right-hand side of 33 and 34, these cannot be evaluated directly for small values of the entropy-regularization ϵ. When $\epsilon \to 0$ we would recover an exact, non entropically-regularized optimal transport – however, this would cause the term inside of the logarithm to exceed numerical precision. For the purpose of introducing the log-domain Sinkhorn algorithm, we make the following definitions. For $\mathbf{1}_{n_i} \in \mathbb{R}_+^{n_i}$ and $\epsilon > 0$, the *Softmin operators* $\mathrm{Smin}_\epsilon^{\mathbf{g}_i}$ are defined for any vectors $\mathbf{f} \in \mathbb{R}^{n_1}$ and $\boldsymbol{\mu} \in \mathbb{R}^{n_2}$ by:

$$\mathrm{Smin}_\epsilon^{\mathbf{1}_{n_1}}(\mathbf{f}) = -\epsilon \log \left(\left\langle e^{-\mathbf{f}/\epsilon}, \mathbf{1}_{n_1} \right\rangle \right) \quad \text{and} \quad \mathrm{Smin}_\epsilon^{\mathbf{1}_{n_2}}(\boldsymbol{\mu}) = -\epsilon \log \left(\left\langle e^{-\boldsymbol{\mu}/\epsilon}, \mathbf{1}_{n_2} \right\rangle \right)$$

Rewriting 34 34 we see:

$$\mathbf{f}_i^{(l+1)} = \frac{\gamma}{\gamma + \epsilon} \left(\epsilon \log \mathbf{g}_{1i} - \epsilon \log \left(\langle e^{-\mathbf{C}_{i,\cdot}/\epsilon}, e^{\boldsymbol{\mu}^{(l)}/\epsilon} \rangle \right) \right) = \frac{\gamma}{\gamma + \epsilon} \left(\epsilon \log \mathbf{g}_{1i} + \mathrm{Smin}_\epsilon^{\mathbf{1}_{n_1}} \left(\mathbf{C}_{ij} - \boldsymbol{\mu}_j \right)_j \right)$$

And:

$$\boldsymbol{\mu}_j^{(l+1)} = \epsilon \log \mathbf{g}_2 - \epsilon \log \left(\langle e^{-\mathbf{C}_{\cdot,j}/\epsilon}, e^{\mathbf{f}^{(l+1)}/\epsilon} \rangle \right) = \epsilon \log \mathbf{g}_{2j} + \mathrm{Smin}_\epsilon^{\mathbf{1}_{n_1}} \left(\mathbf{C}_{ij} - \mathbf{f}_i \right)_i$$

One can simply evaluate the softmin $\mathrm{Smin}_\epsilon^{\mathbf{1}_{n_1}}$ using the log-sum-exp trick, which we broadcast across dimensions for an efficient and stable log-domain update.

S5 Metrics of growth

Let $\mathcal{S}_1 = (\mathbf{S}_1, \mathbf{X}_1)$ and $\mathcal{S}_2 = (\mathbf{S}_2, \mathbf{X}_2)$ be a pair of spatiotemporal tissue slices, corresponding to timepoints t_1 and t_2. For a collection of cell types shared across both slices, enumerated as $\{1, \dots, P\}$, suppose we are given cell type partitions $\mathcal{P}_1 = \{\mathcal{P}_1(p)\}_{p=1}^P$ and $\mathcal{P}_2 = \{\mathcal{P}_2(p)\}_{p=1}^P$ for each slice. The *mass of cell type p at time t_1* is $m_1(p) = |\mathcal{P}_1(p)|$, and the *mass of cell type p at time t_2* is likewise $m_2(p) = |\mathcal{P}_t(p)|$. The *mass-flux of cell type p* over these two timepoints is then given by $m_2(p) - m_1(p)$. Below, we define a metric quantifying how well the cell type mass-flux inferred by an optimal transport method matches the empirical mass-flux of a cell type. If such a method outputs a pair $(\boldsymbol{\Pi}, \boldsymbol{\xi})$ of an alignment matrix and a growth vector, we measure the distortion between $\boldsymbol{\xi}$ and the true mass flux. We discuss the assumptions that go into this metric, and how it can be generalized.

S5.1 A metric of growth: individual cell level

To measure the accuracy of the growth rates across cell types *and* spots within the cell type, we define the true growth rate at time t_1 of cell type p as:

$$\gamma_1(p) = \frac{1}{m_1(p)} \left(\frac{m_2(p) - m_1(p)}{n_1} \right) \tag{35}$$

In calling this a true growth rate, we are making two assumptions: first, that there are no cell type transitions between distinct cell types – in the next subsection we discuss how to relax this. The second assumption made is that the burden of accomplishing the change in mass is shared equally across cells of the same type, which can be viewed as an entropy-maximizing assumption.

The normalization factor $\frac{1}{n_1}$ makes these growth-rates consistent with the slice one mass-normalized values of the marginals $\mathbf{g_1}$, $\mathbf{g_2}$ and ensures consistency across experiments. We quantify the total distortion between the true growth factors for each cell type and those derived from optimal transport as:

$$\mathcal{J}_{growth} = \sum_{p=1}^{P} \sum_{i \in \mathcal{P}_1(p)} \|\boldsymbol{\xi}_i - \gamma_1(p)\|_2^2 \tag{36}$$

This assesses the total divergence between the optimal-transport growth rates and the true, empirical growth rates under a fixed cell type labeling. The sum of the growth-rates across spots within a tissue slice equals the total growth rate of

bioRxiv preprint doi: https://doi.org/10.1101/2024.03.05.583575; this version posted March 10, 2024. The copyright holder for this preprint (which was not certified by peer review) is the author/funder, who has granted bioRxiv a license to display the preprint in perpetuity. It is made available under aCC-BY 4.0 International license.

the slice:

$$\sum_{p=1}^{P} \sum_{i \in \mathcal{P}_1(p)} \gamma_1(p) = \frac{1}{n_1} \sum_{p=1}^{P} \sum_{i \in \mathcal{P}_1^{(p)}} \left(\frac{m_2(p) - m_1(p)}{m_1(p)} \right)$$
$$= \frac{1}{n_1} \sum_{p=1}^{P} (\, m_2(p) - m_1(p) \,)$$
$$= \frac{n_2 - n_1}{n_1}.$$

S5.2 Generalizing the growth-metric to cell type transitions

The definition of growth given in the section above assumes that cells of type p transition and grow to their own cell type, and computes the distortion relative to this assumption. While this is valuable as a baseline metric when no cell type transitions are available, we generalize this notion when we have a density matrix of cell-to-cell reverse-time transitions $\mathbf{T} \in \mathbb{R}_{\geq 0}^{P \times P}$ across P cell types. Supposing we have a vector $\mathbf{m}_2 \in \mathbb{R}_{\geq 0}^{P}$ of cell type masses at time t_2, and likewise $\mathbf{m}_1$ at time t_1, we modify $m_1(p)$ in the definition above to include transitions. The section above implicitly assumes that the matrix is diagonal (in particular, it is the $P \times P$ identity matrix $\mathbf{T} = \mathbb{1}_P$). Under ground truth transition matrix $\mathbf{T}$, the mass of cell type p from time at time t_1 is described in terms of $\mathbf{m}_2$ as follows:

$$m_1(p) = \langle \mathbf{T}_{p,\cdot}, \mathbf{m}_2 \rangle \tag{37}$$

Which is to say, $\mathbf{m}_1$ can be described in terms of $\mathbf{m}_2$ via the matrix-multiplication

$$\mathbf{m}_1 = \mathbf{T}\mathbf{m}_2 \tag{38}$$

and the mass-flux out of cell type p remains as defined in the previous sections with the only adjustment being that the new $m_1(p)$'s account for cell type transitions. As before, we assume the cell type partitions across each slice are consistent with these cell type mass vectors.

Now, consider an alignment matrix $\mathbf{\Pi} \in \mathbb{R}^{n_1 \times n_2}$, and let $\{p \to q\}$ denote the following set:

$$\{p \to q\} = \bigcup_{\substack{i \in \mathcal{P}_1(p) \\ j' \in \mathcal{P}_2(q)}} \{(i, j')\},$$

understood as the event that cell type p of the first slice is mapped to cell type q in the second slice. In making this definition, we show how any alignment matrix $\mathbf{\Pi}$ induces a reverse-time transition matrix: indeed, $\mathbf{\Pi}$ is a positive measure on such pairs, so one can naturally form a coarse-grained $P \times P$ matrix from $\mathbf{\Pi}$ using the events $\{p \to q\}$: first, define

$$\mathbb{R}_{\geq 0}^{P \times P} \ni \overline{\mathbf{\Pi}}, \quad \overline{\mathbf{\Pi}}_{pq} := \mathbf{\Pi}(p \to q)$$

Whatever $\mathbf{T}$ we construct from $\mathbf{\Pi}$ should be a transition matrix, row-normalized such that $\mathbf{1}_P = \mathbf{T}\mathbf{1}_P$. This is achieved by tilting each row p of measure $\overline{\mathbf{\Pi}}$ with the factor $n_1/m_2(q)$:

$$\mathbf{T}^{\star}_{pq} = \left(\frac{n_1}{m_2(q)} \right) \overline{\mathbf{\Pi}}_{pq}. \tag{39}$$

Now, consider the distortion metric defined in (36). Instead of viewing $\mathcal{J}_{growth}$ as a function of $\boldsymbol{\xi}$ only, as was done in the previous section, we now regard it as a function of both $\boldsymbol{\xi}$ and $\boldsymbol{\gamma}_1 \equiv (\gamma_1(p))_{p=1}^{P}$. Just as $\boldsymbol{\xi}$ arises from $\mathbf{\Pi}$, vector $\boldsymbol{\gamma}_1$ is defined from $\mathbf{T}$ through (37) and (35). Given the output $(\mathbf{\Pi}, \boldsymbol{\xi})$ of an OT method, the next proposition gives the optimal pair $(\mathbf{T}, \boldsymbol{\gamma}_1)$ for this output according to the growth distortion metric (36).

Proposition 2. *Given cell type partitions $\mathcal{P}_1$ and $\mathcal{P}_2$ of two SRT slices, let $\mathbf{m}_1$ and $\mathbf{m}_2$ be a pair of cell type mass vectors consistent with these partitions, and related by a transition matrix $\mathbf{T}$ through* (38)*. Fix an alignment matrix $\mathbf{\Pi}$ with $\mathbf{\Pi}^T \mathbf{1}_{n_1} = \frac{1}{n_1}\mathbf{1}_{n_2}$ and consider its associated growth vector $\boldsymbol{\xi} \equiv \boldsymbol{\xi}(\mathbf{\Pi})$. Then, for $\boldsymbol{\gamma}_1 \equiv \boldsymbol{\gamma}_1(\mathbf{T})$, one has*

$$\mathbf{T}^{\star} = \operatorname{argmin}_{(\,\mathbf{T}, \boldsymbol{\gamma}_1(\mathbf{T})\,)} \mathcal{J}_{growth}(\boldsymbol{\xi}, \boldsymbol{\gamma}_1),$$

with $\mathbf{T}^{\star}$ defined from $\mathbf{\Pi}$ in (39).

bioRxiv preprint doi: https://doi.org/10.1101/2024.03.05.583575; this version posted March 10, 2024. The copyright holder for this preprint (which was not certified by peer review) is the author/funder, who has granted bioRxiv a license to display the preprint in perpetuity. It is made available under aCC-BY 4.0 International license.

Proof. When $\boldsymbol{\xi}$ is fixed, the minimizer of 36, in terms of $\boldsymbol{\gamma}_1$, is given in each entry by the cell type sample mean of $\boldsymbol{\xi}$:

$$\gamma_1^\star(p) = \frac{1}{m_1(p)} \sum_{i \in \mathcal{P}_1(p)} \xi_i$$

Thus, we evaluate the right-hand side to find:

$$\frac{1}{m_1(p)} \sum_{i \in \mathcal{P}_1(p)} \xi_i = \frac{1}{m_1(p)} \sum_{i \in \mathcal{P}_1(p)} \left(\boldsymbol{\Pi}_{i,\cdot}^T \mathbf{1}_{n_1} - \frac{1}{n_1} \right) \tag{40}$$

$$= \frac{1}{m_1(p)} \sum_{i \in \mathcal{P}_1(p)} \boldsymbol{\Pi}_{i,\cdot}^T \mathbf{1}_{n_1} - \frac{1}{n_1} \tag{41}$$

$$= \frac{1}{n_1} \frac{m_2(p)}{m_1(p)} - \frac{1}{n_1} \tag{42}$$

Therefore, we have that:

$$\frac{1}{m_1(p)} \sum_{i \in \mathcal{P}_1(p)} \boldsymbol{\Pi}_{i,\cdot}^T \mathbf{1}_{n_1} = \frac{1}{n_1} \frac{m_2(p)}{m_1(p)}$$

And we decompose the following sum according to the cell types p may transition to in the next slice:

$$n_1 \sum_{i \in \mathcal{P}_1(p)} \boldsymbol{\Pi}_{i,\cdot}^T \mathbf{1}_{n_1} = \sum_{q=1}^{P} n_1 \overline{\boldsymbol{\Pi}}_{pq} \tag{43}$$

$$= \sum_{q=1}^{P} m_2(q) \cdot \frac{n_1}{m_2(q)} \overline{\boldsymbol{\Pi}}_{pq} \tag{44}$$

$$\equiv \mathbf{T}_{p,\cdot}^\star \mathbf{m}_2, \tag{45}$$

which shows that the optimal $\gamma_1^\star$ arises from the claimed optimal transition matrix $\mathbf{T}^\star$ defined in (39) We complete the proof by checking that each row of $\mathbf{T}$ sums to one. For notational convenience let $\mathbf{z}_2(q)$ denote the vector in $\mathbb{R}^{n_2}$ whose j'-th component has a 1 if and only if $j' \in \mathcal{P}_2(q)$, and is 0 otherwise; likewise, let $\mathbf{z}_1(p)$ be the vector in $\mathbb{R}^{n_1}$ which is a one-hot encoding of cell type p over the first slice.

$$\sum_{p=1}^{P} \mathbf{T}_{pq} = \frac{n_1}{m_2(q)} \sum_{p=1}^{P} \overline{\boldsymbol{\Pi}}(p \to q) \tag{46}$$

$$= \frac{n_1}{m_2(q)} \sum_{p=1}^{P} \mathbf{z}_1(p)^T \boldsymbol{\Pi} \mathbf{z}_2(q) \tag{47}$$

$$= \frac{n_1}{m_2(q)} \mathbf{1}_{n_1}^T \boldsymbol{\Pi} \mathbf{z}_2(q) \tag{48}$$

The last step is to use the single hard constraint in our semi-relaxed formulation, along with our choice of mass calibration for the second slice: $\boldsymbol{\Pi}^T \mathbf{1}_{n_1} = \frac{1}{n_1} \mathbf{1}_{n_2}$. Continuing from the above display, one has

$$\frac{n_1}{m_2(q)} \left(\boldsymbol{\Pi}^T \mathbf{1}_{n_1} \right)^T \mathbf{z}_2(q) = \frac{n_1}{m_2(q)} \frac{1}{n_1} \mathbf{1}_{n_2}^T \mathbf{z}_2(q) = 1.$$

By definition, all entries of $\mathbf{T}$ are necessarily positive. Therefore these row-sum conditions show us this is indeed a reverse-time transition matrix, and the proof is finished. □

S5.3 Interpretation of per-cell growth distortion $\mathcal{J}_{growth}$

For the setting of $\gamma_1(p)$ in the previous section, rather than matching the cell type mass-fluxes assuming cell types transitioning to themselves, we find the optimal matrix $\mathbf{T}$ of cell types transitioning from t_1 to t_2, calculate the adjusted

bioRxiv preprint doi: https://doi.org/10.1101/2024.03.05.583575; this version posted March 10, 2024. The copyright holder for this preprint (which was not certified by peer review) is the author/funder, who has granted bioRxiv a license to display the preprint in perpetuity. It is made available under aCC-BY 4.0 International license.

mass contributed to $\mathbf{m}_2$ from cell type p at t_1 as $m_1(p) = \mathbf{T}_{p,\cdot}\mathbf{m}_2$, and calculate the expected distortion in the mass-flux. As noted in 2, the minimizer of $\mathcal{J}_{growth}$ over $\boldsymbol{\gamma}_1$ is simply the sample mean of the per-cell type mass-flux, given as:

$$\gamma_1(p) = \frac{1}{m_1(p)} \sum_{i \in \mathcal{P}_1(p)} \xi_i.$$

Therefore an alternative interpretation to one in which we match the mass-flux of the optimal transition matrix is that we are measuring the sum of the variances of each mass-flux variable within each cell type:

$$\sum_{\text{(cell types) } p=1}^{P} \mathbf{Var}\left(\boldsymbol{\xi}_i \mid i \in \mathcal{P}_1(p)\right)$$

In a tissue or cell type, the assumption that growth is relatively homogeneous or smoothly varying, as opposed to coming from a very small, sparse, set of cells, has been observed in morphogen studies where within tissues like the imaginal wing disk in drosophila one observes uniform cell divisions and growth [15] [25] [27]. As such, measuring the within-cell type variance of the growth-factors is biologically reasonable and captures whether growth patterns are very sparse (i.e. when aligned via an unbalanced routine which assigns a few "representative" cells as those which grow), or whether growth patterns are relatively consistent.

S6 Procrustes-like problems and a cell migration metric

S6.1 Quantifying a metric of naive rigid-body migration

Suppose we are given the solution to either the generalized Procrustes' problem for an orthogonal transformation $\mathbf{Q} \in \mathrm{O}(n)$ or the generalized Wahba's problem for a rotation $\mathbf{Q} \in \mathrm{SO}(n)$ along with a general translation vector $\mathbf{h} \in \mathbb{R}^n$ as described in S6.3. This then describes a simple rigid-body transformation which relates the coordinate frames of slice 1 and 2, $\mathcal{S}_1 \approx \tilde{\mathcal{S}}_2 = \{\mathbf{Q}\left(\mathbf{s}'_{j'} - \mathbf{h}\right) : \mathbf{s}'_{j'} \in \mathcal{S}_2\}$. Supposing the true transformation relating the two frames is indeed a rigid-body transformation, one may directly quantify the cost of an alignment with respect to the matrix $\mathbf{\Pi}$ given $\mathbf{Q}, \mathbf{h}$. We introduce this as our *naive rigid-body migration* metric, given as:

$$\sum_{i=1}^{n_1} \left(\mathbf{\Pi}_{i,\cdot}^T \mathbf{1}_{n_2}\right)^{-1} \left(\sum_{j'=1}^{n_2} \mathbf{\Pi}_{ij'} \|\mathbf{s}_i - \mathbf{Q}\left(\mathbf{s}'_{j'} - \mathbf{h}\right)\|_2^2 \right) \tag{49}$$

Where we take the difference between the spatial points $\mathbf{s}_i$ and $\mathbf{s}'_{j'}$ in our pair of slices $(\mathbf{X}^{(1)}, \mathbf{S}^{(1)})$ and $(\mathbf{X}^{(2)}, \mathbf{S}^{(2)})$. This difference is computed under the posterior implied by $\mathbf{\Pi}$, and as we compare matrices $\mathbf{\Pi}$ which may be balanced, unbalanced, or semi-relaxed, we normalize by a factor of $\mathbf{\Pi}_{i,\cdot}^T \mathbf{1}_{n_2}$ so that any posterior in 49 is normalized to a distribution consistently. We call this metric *naive*, as it is clear this transformation has determinant 1, and neither allows for volume expansion (growth) or volume shrinkage (death). Moreover, the true transformation underlying tissue development might involve some arbitrary (e.g. diffeomorphic) transformation, such that the rigid-body assumption is more generally inappropriate. As such, it is not necessarily the case that lower loss under this metric is universally better. However, one can say that if the alignment tends to incur exceptionally high loss under this metric, without any geometric consistency, the alignment may be spatially unrealistic. In other words, it might be aligning the sub-level set of points with similar features $\Omega_{\mathbf{x}'_{j'}} = \{\mathbf{s}_i \in \mathcal{S}_1 : \|\mathbf{s}_i - \mathbf{s}'_{j'}\|_2^2 \leq (\text{const.})\}$ without accounting for the geometry of either slice.

S6.2 Invariance of the method to rigid-body transformations, or group action by members of SE(2)

Let us call each spatial point in the first slice $\mathbf{s}_i \in \mathbb{R}^2$, and each point in the second slice $\mathbf{s}'_{j'} \in \mathbb{R}^2$. Suppose we want the second slice to be in the same coordinate basis as the first slice, but we do not know $\mathbf{Q} \in \mathrm{SE}(2), \mathbf{h} \in \mathbb{R}^2$, representing the rigid-body transformation of the data $\tilde{\mathbf{s}}_{j'} = \mathbf{Q}(\mathbf{s}'_{j'} - h)$ which moves us into the correct, shared coordinate-frame. A reasonable question to ask is whether our objective returns an alignment matrix $\mathbf{\Pi}$ which is unique, independent of the specific $\mathbf{Q}$, $\mathbf{h}$ that relate the two coordinate frames. From the general objective in 20, it is clear that our objective only depends on the points in the first and second slices $\mathbf{s}_i$, $\mathbf{s}'_{j'}$ through the distance matrices $\mathbf{D}^{(1)}$ and $\mathbf{D}^{(2)}$. Considering that $\mathbf{s}_i$ is in the correct basis, let us restrict our attention to $\mathbf{s}'_{j'}$ and $\mathbf{D}^{(2)}$. It is simple to check that:

$$[\mathbf{D}^{(2)}]_{i'j'} = \|\mathbf{s}'_{i'} - \mathbf{s}'_{j'}\|_2 = \|\mathbf{Q}\mathbf{s}'_{i'} - \mathbf{Q}\mathbf{s}'_{j'}\|_2 = \|\tilde{\mathbf{s}}_{i'} - \tilde{\mathbf{s}}_{j'}\|_2$$

bioRxiv preprint doi: https://doi.org/10.1101/2024.03.05.583575; this version posted March 10, 2024. The copyright holder for this preprint (which was not certified by peer review) is the author/funder, who has granted bioRxiv a license to display the preprint in perpetuity. It is made available under aCC-BY 4.0 International license.

Which implies that our objective does not depend on the spatial location or rotation of the second slice relative to the first. Uniqueness therefore only depends on the convexity of the objective, which could in principle be achieved by using the projection of $\mathbf{D}^{(1)}, \mathbf{D}^{(2)}$ onto the vector space of positive-semi definite matrices $\mathcal{S}_n^+$ or kernelization.

The next reasonable question is whether one can recover the correct $\mathbf{Q}, \mathbf{h}$ given the alignment matrix $\mathbf{\Pi}$. Problems of this form include the Orthogonal Procrustes problem (where $\mathbf{Q}$ is simply an orthogonal matrix), and Wahba's problem (where $\mathbf{Q} \in \mathrm{SO}(2)$ explicitly), which are both well studied and have existing solution methods. Thus, the final problem needed to align the coordinate frame can be given (in Procrustes' form) as:

$$\begin{aligned} \min_{\mathbf{Q}\in\mathbb{R}^{2\times 2},\, \mathbf{h}\in\mathbb{R}^2} \quad & \sum_{i,j'} \mathbf{\Pi}_{ij'} \|\mathbf{s}_i - \mathbf{Q}(\mathbf{s}'_{j'} - \mathbf{h})\|_2^2 \\ \text{s.t.} \quad & \mathbf{Q}^{\mathbf{T}}\mathbf{Q} = \mathbb{1}_2 \end{aligned} \tag{50}$$

Or, in Wahba's problem form:

$$\min_{\mathbf{Q}\in\mathrm{SO}(2),\, \mathbf{h}\in\mathbb{R}^2} \quad \sum_{i,j'} \mathbf{\Pi}_{ij'} \|\mathbf{s}_i - \mathbf{Q}(\mathbf{s}'_{j'} - \mathbf{h})\|_2^2 \tag{51}$$

We offer both the Procrustes' and Wahba's form, with the latter having the restriction that $\mathbf{Q} \in \mathrm{SO}(2)$ requires $\det \mathbf{Q} = 1$, which is to say we seek an explicit (orientation-preserving) rotation rather than a general orthogonal transformation (which may be a rotation composed with a reflection). In either case, the problem of finding a correct alignment is something that emerges *after* the outputs of `DeST-OT`, which is itself completely independent of the coordinate frame the first and second slice are set in and is invariant to linear translation and rotation, unlike works involving Gaussian processes such as [18].

S6.3 Review of the Solution to Wahba's problem

First, we consider an appropriate transformation of the coordinates to eliminate the dependence on the translation, for this light generalization of Wahba's problem to a joint-distribution across both slices [10]. In particular, consider the change of variables given by:

$$\mathbf{h} = -\mathbf{Q}^T \mathbf{s}_{\text{cm}} + \mathbf{s}'_{\text{cm}} + \boldsymbol{\nu}$$

For $\mathbf{s}_{\text{cm}}, \mathbf{s}'_{\text{cm}}$ representing the center of mass of slice 1 and slice 2, and $\boldsymbol{\nu}$ an undetermined constant. In particular,

$$\mathbf{s}_{\text{cm}} = \left(\mathbf{1}_{n_1}^T \mathbf{\Pi} \mathbf{1}_{n_2}\right)^{-1} \sum_{i=1}^{n_1} \sum_{j'=1}^{n_2} \mathbf{\Pi}_{ij'} \mathbf{s}_i, \qquad \mathbf{s}'_{\text{cm}} = \left(\mathbf{1}_{n_1}^T \mathbf{\Pi} \mathbf{1}_{n_2}\right)^{-1} \sum_{i=1}^{n_1} \sum_{j'=1}^{n_2} \mathbf{\Pi}_{ij'} \mathbf{s}'_{j'} \tag{52}$$

Substituting this into the primal objective:

$$\begin{aligned} \sum_{i,j'} \mathbf{\Pi}_{ij'} \|\mathbf{s}_i - \mathbf{Q}(\mathbf{s}'_{j'} - \mathbf{h})\|_2^2 &= \sum_{i,j'} \mathbf{\Pi}_{ij'} \|\mathbf{s}_i - \mathbf{Q}(\mathbf{x}'_{j'} - (-\mathbf{Q}^T \mathbf{s}_{\text{cm}} + \mathbf{s}'_{\text{cm}} + \boldsymbol{\nu}))\|_2^2 \\ &= \|\boldsymbol{\nu}\|_2^2 (\mathbf{1}_{n_1}^T \mathbf{\Pi} \mathbf{1}_{n_2}) + \sum_{i,j'} \mathbf{\Pi}_{ij'} \|(\mathbf{s}_i - \mathbf{s}_{\text{cm}}) - \mathbf{Q}(\mathbf{s}'_{j'} - \mathbf{s}'_{cm})\|_2^2 \\ &+ \boldsymbol{\nu}^T \left(\left(\sum_{i,j'} \mathbf{\Pi}_{ij'} \mathbf{s}_i - (\mathbf{1}_{n_1}^T \mathbf{\Pi} \mathbf{1}_{n_2}) \mathbf{s}_{\text{cm}} \right) + \left(\sum_{i,j'} \mathbf{\Pi}_{ij'} \mathbf{s}'_{j'} - (\mathbf{1}_{n_1}^T \mathbf{\Pi} \mathbf{1}_{n_2}) \mathbf{s}'_{\text{cm}} \right) \right) \end{aligned}$$

By definition of the center of mass-coordinates, the above equates to the following, with the minimization over $\mathbf{r}$ now over $\boldsymbol{\nu}$:

$$\min_{\mathbf{Q}\in\mathrm{SO}(2),\, \boldsymbol{\nu}\in\mathbb{R}^2} \quad \|\boldsymbol{\nu}\|_2^2 (\mathbf{1}_{n_1}^T \mathbf{\Pi} \mathbf{1}_{n_2}) + \sum_{i,j'} \|(\mathbf{s}_i - \mathbf{s}_{\text{cm}}) - \mathbf{Q}(\mathbf{s}'_{j'} - \mathbf{s}'_{\text{cm}})\|_2^2 \tag{53}$$

Where the independence of the left and right primal objectives yields that the minimizer is simply $\boldsymbol{\nu}^\star = \mathbf{0}$. After the optimization for the rotation, the optimal translation is simply: $\mathbf{h}^\star = -(\mathbf{Q}^\star)^T \mathbf{s}_{\text{cm}} + \mathbf{s}'_{\text{cm}}$. Thus, one may simply focus on the problem:

$$\min_{\mathbf{Q}\in\mathrm{SO}(2)} \quad \sum_{i,j'} \mathbf{\Pi}_{ij'} \|(\mathbf{s}_i - \mathbf{s}_{\text{cm}}) - \mathbf{Q}(\mathbf{s}'_{j'} - \mathbf{s}'_{\text{cm}})\|_2^2 \tag{54}$$

bioRxiv preprint doi: https://doi.org/10.1101/2024.03.05.583575; this version posted March 10, 2024. The copyright holder for this preprint (which was not certified by peer review) is the author/funder, who has granted bioRxiv a license to display the preprint in perpetuity. It is made available under aCC-BY 4.0 International license.

The presence of the joint distribution keeps this from being set in the standard form which Wahba's problem is presented and solved in. We give a simple equivalent form, introducing the center-shifted coordinate matrices across spatial locations in the slices:

$$\mathbf{S}^{(1)}_{\text{cm}} = \begin{pmatrix}\mathbf{s}_1 - \mathbf{s}_{\text{cm}} & \dots & \mathbf{s}_{n_1} - \mathbf{s}_{\text{cm}}\end{pmatrix}$$
$$\mathbf{S}^{(2)}_{\text{cm}} = \begin{pmatrix}\mathbf{s}'_{1'} - \mathbf{s}'_{\text{cm}} & \dots & \mathbf{s}'_{n'_2} - \mathbf{s}'_{\text{cm}}\end{pmatrix}$$

And consider the equivalent objective, with vec denoting the vectorization operation which column-stacks a matrix, $\mathbf{\Pi}^{1/2}$ denoting the element-wise square-root of the entries of $\mathbf{\Pi}$, and $\odot$ denoting the elementwise, or Hadamard, product:

$$\min_{\mathbf{Q}\in \text{SO}(2)} \left\| \left(\mathbf{1}_2 \operatorname{vec}\left((\mathbf{\Pi}^{1/2})^T\right) \odot \left(\mathbf{S}^{(1)}_{\text{cm}} \otimes \mathbf{1}^T_{n_2}\right)\right) - \mathbf{Q}\left(\mathbf{1}_2 \operatorname{vec}\left((\mathbf{\Pi}^{1/2})^T\right) \odot \left(\mathbf{1}^T_{n'} \otimes \mathbf{S}^{(2)}_{\text{cm}}\right)\right)\right\|_F^2 \quad (55)$$

We rename the matrices to be concise as $\mathbf{G}^{(1)}$, $\mathbf{G}^{(2)}$:

$$\min_{\mathbf{Q}\in \text{SO}(2)} \quad \|\mathbf{G}^{(1)} - \mathbf{Q}\mathbf{G}^{(2)}\|_F^2 \quad (56)$$

The simple solution [10] can be given using an SVD of $\mathbf{G}^{(1)}(\mathbf{G}^{(2)})^T = \mathbf{U\Sigma V}^T$. Where the final orthogonal transformation would be given as $\mathbf{Q} = \mathbf{UV}^T$. If one adds the restriction that $\det\left(\mathbf{UV}^T\right) = 1$ for a rotation, one would find $\mathbf{Q}$ as:

$$\mathbf{Q} = \mathbf{U}\operatorname{diag}\begin{pmatrix}1 & \det\left(\mathbf{UV}^T\right)\end{pmatrix}\mathbf{V}^T.$$

S7 Generate feature (expression) vectors for simulated data

S7.1 Generate feature vectors for simulated 1D slices

Our simplest experiment considers a pair of *one-dimensional* tissue slices with the same number of spots (51 total). Writing N in place of 25 for clarity, the spatial coordinates of the first and second slices are given by identical matrices, namely $\mathbf{S}^{(1)} \equiv \mathbf{S}^{(2)} = \begin{bmatrix}-N & -(N-1) & \dots & -1 & 0 & 1 & \dots & (N-1) & N\end{bmatrix}$, understood as a column vector. Synthetic features are constructed by first assigning one of two cell type labels ("A" or "B") to each spot, and then assigning numerical features based on cell type. As in 2.3.1, cell types partition each domain, we write these as $\mathcal{P}_1$ and $\mathcal{P}_2$, where:

$$\mathcal{P}_1(\text{A}) = \{-N, -N+1, \dots w_1 - 1, w_1\}, \quad \mathcal{P}_1(\text{B}) = \{w_1 + 1, \dots, N\}\}$$
$$\mathcal{P}_2(\text{A}) = \{-N, -N+1, \dots w_2 - 1, w_2\}, \quad \mathcal{P}_2(\text{B}) = \{w_2 + 1, \dots, N\}, \}$$

setting $w_1 = -10$ and $w_2 = 5$ in the experiment to be the "pivots" marking a change in cell type.

We generate eight-dimensional feature by sampling random vectors $\boldsymbol{v}_1, \boldsymbol{v}_2, \boldsymbol{v}_3, \boldsymbol{v}_4$ independently and uniformly from the unit sphere of $\mathbb{R}^4$. Let $\mathbf{0} \in \mathbb{R}^4$ be the zero vector, and let $\circ$ denote concatenation of vectors. We set $\tilde{\boldsymbol{v}}_1 := \boldsymbol{v}_1 \circ \mathbf{0}$, $\tilde{\boldsymbol{v}}_2 := \boldsymbol{v}_2 \circ \mathbf{0}$, $\tilde{\boldsymbol{v}}_3 := \mathbf{0} \circ \boldsymbol{v}_3$ and $\tilde{\boldsymbol{v}}_4 := \mathbf{0} \circ \boldsymbol{v}_4$. Over cell type A, we generate features by linearly interpolating feature $\tilde{\boldsymbol{v}}_1$ at spot $-N$ with feature $\tilde{\boldsymbol{v}}_2$ at spot w_1 or w_2, depending on the slice. Over cell type B, we generate features by linearly interpolating feature $\tilde{\boldsymbol{v}}_3$ at either w_1 or w_2, with feature $\tilde{\boldsymbol{v}}_4$ at spot N. Thus, each cell type is characterized by a feature gradient in four dimensions, and the features at distinct cell types are orthogonal by design.

S7.2 Generate feature vectors for simulated 2D slices

We generalize the above by defining a two-dimensional model of tissue slices. Let E denote the centered, circular ellipse of radius $r = 25$:

$$E = \left\{\mathbf{s} \equiv (x, y) \in \mathbb{R}^2 : \frac{x^2}{r^2} + \frac{y^2}{r^2} \leq 1\right\}$$

Let $\mathbb{T}$ be the subset of the integer square lattice $\mathbb{Z}^2$ consisting of all integer pairs whose sum is odd: $\mathbb{T} = \{(x, y) \in \mathbb{Z}^2 : (x + y) \bmod 2 = 1\}$. Then $\mathbb{T}$ is a triangular lattice, mimicking the spatial organization of SRT data output by the Visium platform. Let $\mathbf{S}^{(1)} = \mathbf{S}^{(2)} = \mathbf{S}$ denote the stack of spatial coordinates associated to the set $E \cap \mathbb{T}$.

bioRxiv preprint doi: https://doi.org/10.1101/2024.03.05.583575; this version posted March 10, 2024. The copyright holder for this preprint (which was not certified by peer review) is the author/funder, who has granted bioRxiv a license to display the preprint in perpetuity. It is made available under aCC-BY 4.0 International license.

As above, we use a simple spatial condition to partition each slice into two pieces. Here, we set two "pivot" lines, given by $y = w_1$ and $y = w_2$. The cell type partitions $\mathcal{P}_1, \mathcal{P}_2$ over the two slices are defined as follows:

$$\mathcal{P}_1(\mathrm{A}) = \{\mathbf{s} \equiv (x, y) \in E \cap \mathbb{T} : y > w_1\}, \quad \mathcal{P}_1(\mathrm{B}) = \{\mathbf{s} \equiv (x, y) \in E \cap \mathbb{T} : y \leq w_1\}$$
$$\mathcal{P}_2(\mathrm{A}) = \{\mathbf{s} \equiv (x, y) \in E \cap \mathbb{T} : y > w_2\}, \quad \mathcal{P}_2(\mathrm{B}) = \{\mathbf{s} \equiv (x, y) \in E \cap \mathbb{T} : y \leq w_2\}$$

for $w_1, w_2 = \pm 10$.

We assign features in a similar manner to the one-dimensional case. Previously we assigned features to the boundary of cell type segments and linearly interpolated between these.

In this slightly more general setting, let $R_i(\mathrm{A})$ and $R_i(\mathrm{B})$ denote (smallest) bounding rectangles for sets $\mathcal{P}_i(\mathrm{A})$ and $\mathcal{P}_i(\mathrm{B})$ for slices $i = 1, 2$.

We assign the first four unit vectors in the standard basis $(\mathbf{e}_j)_{j=1}^8$ of $\mathbb{R}^8$ to cell type A, and the last four to cell type B. The top and bottom sides of $R_i(\mathrm{A})$ are assigned to features $\mathbf{e}_1$ and $\mathbf{e}_3$, while the left and right sides of the rectangle are assigned to features $\mathbf{e}_2$ and $\mathbf{e}_4$. We make similar assignments for $R_i(\mathrm{B})$ using the last four basis vectors. At each spot $\mathbf{s}$, lying in some bounding rectangle R for its cell type, the feature assigned to $\mathbf{s}$ is a convex combination of the features decorating the top and bottom sides of R, plus the features decorating the left and right sides of R. In particular, for $R = [x_{\min}, x_{\max}] \times [y_{\min}, y_{\max}]$ and $(x, y) \in R$, the coefficients λ_x, λ_y are defined as:

$$\lambda_x = \frac{x - x_{\min}}{x_{\max} - x_{\min}}, \qquad \lambda_y = \frac{y - y_{\min}}{y_{\max} - y_{\min}}$$

Thus, for a given cell type we have two gradients along the x and y direction which are scaled equivalently between the two slices $\mathbf{S}^{(1)}$ and $\mathbf{S}^{(2)}$ which we seek to align. The final feature vector within cell type A is therefore given as

$$\mathbf{f}_A(x, y) = \lambda_x \mathbf{f}_{x,L} + (1 - \lambda_x)\mathbf{f}_{x,R} + \lambda_y \mathbf{f}_{y,T} + (1 - \lambda_y)\mathbf{f}_{y,B}$$

and repeat this for cell type B as

$$\mathbf{g}_B(x, y) = \lambda_x \mathbf{g}_{x,L} + (1 - \lambda_x)\mathbf{g}_{x,R} + \lambda_y \mathbf{g}_{y,T} + (1 - \lambda_y)\mathbf{g}_{y,B}$$

choosing features which are mutually-orthogonal–for simplicity 8-dimensional unit vectors $\mathbf{e}_{1:8}$. Between $\mathbf{S}^{(1)}$ and $\mathbf{S}^{(2)}$ these features are consistent, and λ_x, $\lambda_y \in [0, 1]$ gives the proportion of each feature depending on how far the (x, y) coordinate is along the axis of each ellipse within a given cell type. I.e. for $(x_1, y_1) \in \mathbf{S}^{(1)}$, $(x_2, y_2) \in \mathbf{S}^{(2)}$ we have that if $\lambda_{x_1}^A = \lambda_{x_2}^A$ and $\lambda_{y_1}^A = \lambda_{y_2}^A$ then $\mathbf{f}_A(x_1, y_1) = \mathbf{f}_A(x_2, y_2)$ and the two features should be aligned between timepoints 1 and 2 (likewise for cell type B). As the features are chosen to be orthogonal, this alignment is unique. To model noise, we assume that the data we observe at each point (x, y) is distributed as $\sim \mathcal{N}(\mathbf{f}_A(x, y), \sigma^2 \mathbb{1}_8)$ for cell type A and $\sim \mathcal{N}(\mathbf{g}_B(x, y), \sigma^2 \mathbb{1}_8)$ for cell type B for varying levels of σ.

S8 Benchmarking methods

PASTE [45] By design, PASTE cannot align spatiotemporal data and does not infer cell growth and death because it uses balanced OT. For the purpose of benchmarking experiments in this work, we relax PASTE's balanced constraints so that we optimize the unbalanced version of the PASTE objective. For the alignment matrix $\mathbf{\Pi}$ computed by relaxed PASTE, we then take the difference between the row sums of $\mathbf{\Pi}$ and the uniform distribution $\mathbf{g}_1$ (Eq. (4)) as PASTE's inferred growth $\boldsymbol{\xi}$.

moscot [21] We use `moscot.problems.spatiotemporal.SpatioTemporalProblem` for solving spatiotemporal alignment problems in this work using moscot. For the alignment matrix $\mathbf{\Pi}$ computed by moscot, we take the difference between the row sums of $\mathbf{\Pi}$ and the uniform distribution $\mathbf{g}_1$ as moscot's inferred growth $\boldsymbol{\xi}$. For benchmarking experiments related to growth *rates* instead of growth (§**??**), we use the numbers returned by `SpatioTemporalProblem.posterior_growth_rates` as moscot's inferred growth *rates* over spots in timepoint t_1.

SLAT [44] We use `scSLAT.model.run_SLAT` of the scSLAT package for computing an embedding for each spot in each timepoint. We then use `scSLAT.model.spatial_match` to compute an alignment between spots across the two timpoints. We construct a matrix $\mathbf{\Pi}$ such that $\mathbf{\Pi}_{ij'} = 1$ if spot i in timepoint 1 is the best spot aligned to spot j' in timepoint 2. We then divide $\mathbf{\Pi}$ by the number of spots at timpoint 1 to convert it into a semi-relaxed transport matrix. We take the difference between the row sums of $\mathbf{\Pi}$ and the uniform distribution $\mathbf{g}_1$ as SLAT's inferred growth $\boldsymbol{\xi}$.

bioRxiv preprint doi: https://doi.org/10.1101/2024.03.05.583575; this version posted March 10, 2024. The copyright holder for this preprint (which was not certified by peer review) is the author/funder, who has granted bioRxiv a license to display the preprint in perpetuity. It is made available under aCC-BY 4.0 International license.

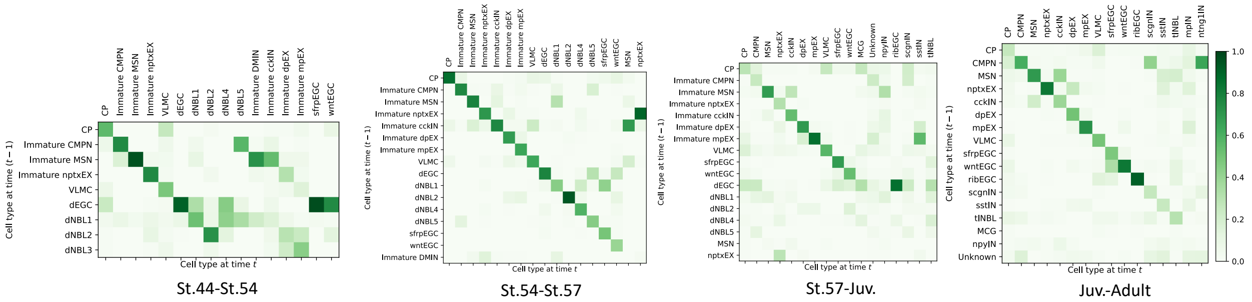

Figure S1: Cell type transition matrix at each pair of timepoints during axolotl brain development Rows are cell types at the previous timepoint. Columns are cell types at the next timepoint. Matrices are column normalized.

STalign [8] We first "rasterize" the spatial positions of our input data into two greyscale images. This effectively convolves a scatterplot of the spatial data with two-dimensional Gaussian noise, to produce an image approximating each tissue slice. We then ran STalign's `LDDMM` on the pair of images, outputting a diffeomorphism φ (which maps the first slice to the second) and its inverse φ^{-1} (mapping the second slice to the first). We then use φ^{-1} to construct a transport plan $\mathbf{\Pi}$ as follows: for spot j' in the second slice, we apply φ^{-1} to $s_{j'}$, and select s_i in the first slice which is closest to $\varphi^{-1}(s_{j'})$. Then we set $\mathbf{\Pi}_{ij'} = 1$, and repeat this procedure for each spot in the second slice, filling out each column of $\mathbf{\Pi}$. The transport plan is thus a deterministic map from the spots of the second slice into those of the first. We then divide $\mathbf{\Pi}$ by the number of spots at timpoint 1 to convert it into a semi-relaxed transport matrix. We take the difference between the row sums of $\mathbf{\Pi}$ and the uniform distribution $\mathbf{g}_1$ as STalign's inferred growth $\boldsymbol{\xi}$.
